# Greater climate change adaptation potential in populations of *Quercus macrocarpa* at edges of latitudinal gradient

**DOI:** 10.1111/nph.71003

**Published:** 2026-02-16

**Authors:** Lucy M. S. Rea, Laura Ostrowsky, Rebekah A. Mohn, Mira Garner, Lindsey Worcester, Cathleen Lapadat, Heather R. McCarthy, Andrew L. Hipp, Jeannine Cavender Bares

**Affiliations:** ^1^ Department of Ecology, Evolution, and Behavior College of Biological Sciences, University of Minnesota 140 Gortner Laboratory, 1479 Gortner Ave St Paul MN 55108 USA; ^2^ Department of Organismic and Evolutionary Biology Harvard University 26 Oxford St Cambridge MA 02138 USA; ^3^ Science and Conservation, The Morton Arboretum 4100 Illinois Route 53 Lisle IL 60532 USA; ^4^ Department of Plant and Microbial Biology College of Biological Sciences, University of Minnesota 140 Gortner Laboratory, 1479 Gortner Ave St Paul MN 55108 USA; ^5^ Pritzker Lab, The Field Museum 1400 S Lake Shore Dr Chicago IL 60605 USA; ^6^ School of Biological Sciences, University of Oklahoma Norman OK 73019 USA

**Keywords:** adaptation potential, bur oak, climate adaptation, common garden, genetic variance, heritability, *Quercus macrocarpa*, selection

## Abstract

- With current climate trajectories, tree populations will encounter novel selection pressures that risk local extinction if they are unable to acclimate or adapt. Within a reciprocal transplant experiment with *Quercus macrocarpa* L. established across a latitudinal gradient, we asked: (1) Is there genetic variation within populations? (2) Are there differences in the direction and strength of selection? (3) Do traits within populations differ in adaptation potential in response to future climate conditions?
- Within each population in each of three gardens (Minnesota, Illinois, and Oklahoma), we estimated genetic variance for nine traits grouped in three realms: physiology, spectral reflectance features, and morphology/growth. We also analyzed selection on these traits and assessed their potential adaptive response to selection.
- Our results indicate that traits related to morphology and growth have high genetic variance and are under strong directional selection in warmer gardens. The populations that represent extreme ends of the climatic gradient have high potential to adapt to climate change, based on their responses to selection in the warmest garden (Oklahoma).
- These results inform strategies to improve species resilience by providing seed source information relevant to managers planning assisted migration to promote climate change adaptation.

## Introduction

Ongoing climate change is introducing novel conditions that will impact tree species' ranges. With shifting climatic conditions, populations of trees may need to migrate to higher latitudes, adapt to novel conditions, or risk local extinction (Aitken *et al*., 2008). The rate of climate change may outpace the rate at which trees can evolve in response to climate change (Shaw & Etterson, 2012; Franks *et al*., 2014). Furthermore, different regions are expected to experience different rates of warming, for example higher rates of warming are expected in the northern United States, particularly in winter, relative to southern regions (Handler *et al*., 2014; Young & Young, 2025). However, increased warming and drought in already hot southern climates can intensify physiological stress (Vollenweider & Günthardt‐Goerg, 2005; Barnabás *et al*., 2008; Li *et al*., 2024). Understanding the ability of populations to adapt and determining the lag in adaptation to climate change is critical to management responses such as assisted migration or land conservation to protect at‐risk populations (Aitken & Whitlock, 2013; Etterson *et al*., 2020).

High levels of existing genetic diversity in populations can improve the likelihood of the population adapting to ongoing climate change. Populations that are larger and more connected have more opportunities for gene flow between demes (groups of connected individuals within a population) and greater genetic variance than less connected populations with lower gene flow (Schlaepfer *et al*., 2018). These larger, more connected populations are likely to have higher effective population sizes and levels of genetic variance, and thus higher adaptive response rate when encountering strong selective pressure (Willi *et al*., 2006; Alberto *et al*., 2013; Hoffmann *et al*., 2017). A larger population increases the odds that individuals suited to novel climate or environmental conditions will already exist within the population, enabling them to persist (Vandewoestijne *et al*., 2008; Ahrens *et al*., 2020). The response to selection relies on the proportion of phenotypic variation that is due to genetics, or the heritability (Falconer & Mackay, 1996, p. 210–211). Populations that experience variable environments and are under strong selection pressure (e.g. due to seasonal drought) may have high genetic variance because opposing phenotypes are selected for in different years (Meireles *et al*., 2017; Cavender‐Bares, 2019; Ramírez‐Valiente *et al*., 2019). Selection in a population for traits that improve adaptation to climate change is critical for populations to persist.

Deciphering which traits will be selected for with ongoing climate change is critical to predicting the eventual fate of tree populations. Genetic variation may be hidden in a population until exposed to novel conditions (Bemmels & Anderson, 2019), which may result in different physiological responses than populations expressed in the past. Increased drought and aridity are expected with the current warming trends in the global climate (Sheffield & Wood, 2008; Intergovernmental Panel on Climate Change (IPCC), 2022). Consequently, different physiological trait values may be selected for under climate change. For example, increased aridity induced by climate change may select for individuals with greater ability to tolerate or avoid drought damage. Dry conditions make the ability to retain water in the leaves important for survival due to the depletion of water pools in the soil and thus the plant, leading to eventual vessel embolism (McDowell *et al*., 2022). Hydrated leaves are also important for photosynthesis rates by maintaining stomatal opening for carbon assimilation and providing the necessary hydrogen for the photosynthetic process (Cornic, 2000; Lawlor, 2002; Wang *et al*., 2022). Maintaining sufficient photosynthetic rates is critical to providing the carbon used for growth and stress responses to drought, cold, and herbivory (McDowell *et al*., 2022). Photosynthetic capacity can be increased with high foliar Chl concentrations through increased light energy capture (Govindjee, 2004; Croft *et al*., 2017), while photoprotective pigments, including carotenoids (such as xanthophyll pigments) or anthocyanins, can help to prevent photodamage by absorbing excess light in plants that have lower photocapacity (Adams & Demmig‐Adams, 1995; Gould, 2004; Demmig‐Adams & Adams III, 2006; Ashikhmin *et al*., 2023). In more arid or colder climates, these pigments tend to be prevalent in greater concentrations (pool sizes) and to convert to active forms in the leaves, providing an adaptive advantage by increasing photoprotection in stressful climates (Wujeska *et al*., 2013; Verhoeven, 2014; Ramírez‐Valiente *et al*., 2015).

Measuring physiological traits on a plant often requires destructive sampling, which is not a preferred option in long‐term experiments. Spectral reflectance measurements are a nondestructive means of gathering information about plant phenotypes (Kothari & Schweiger, 2022; Cavender‐Bares *et al*., 2025). Reflectance spectra are measures of the light reflected from the surface of the leaf from the visible to the shortwave infrared region of the light spectrum (400–2400 nm) (Jacquemoud & Ustin, 2019). The amount of light absorbed or reflected by the leaf in different regions of the spectra can vary depending on features of the leaf, such as pigment quantity, leaf thickness, and leaf water content (Cavender‐Bares *et al*., 2017; Serbin & Townsend, 2020). The changes in spectral reflectance at specific wavelengths can be informative on their own as genetically based markers to distinguish among populations, and the relationships between certain wavelengths can be used as spectral indices for leaf water content and leaf pigments (Sims & Gamon, 2002, 2003; Gamon *et al*., 2019; Jacquemoud & Ustin, 2019; Corbin *et al*., 2025). In common garden experiments where individuals come from a known genetic lineage, spectral phenotypes can thus be used to quantitatively assess the genetic basis for similarities or differences in phenotypes within and among plant populations. We can assess if current populations have spectral traits that may be selected for or against under changing environments using associations in the spectra with certain plant functions or traits.

In our study, we used a reciprocal transplant experiment with three populations of the widespread tree species *Quercus macrocarpa* L. (bur oak) established across a latitudinal gradient to quantify genetic variation and to test for evidence of selection on spectral traits among populations and across latitudes. Long‐lived, wind‐pollinated trees such as *Q. macrocarpa* are expected to have high genetic variation that could be beneficial in the face of ongoing climate change (Hamrick, 2004; McDowell *et al*., 2008). We used the latitudinal gradient of our three gardens as a proxy for expected climate change, with the southernmost garden representing expected future climate conditions in northern latitudes. Our overarching goal was to determine the potential for *Q. macrocarpa* populations to evolve in response to ongoing change trajectories. In pursuit of this goal, we asked three central questions: (Q1) Is there genetic variation within populations for traits relevant to climate change? (Q2) Are there differences in direction and strength of selection for traits in each garden? Specifically, is the strength of selection stronger for northern populations in southern gardens compared to their home gardens and compared to selection on the southern population? (Q3) Do traits relevant to climate change within populations have the potential to adapt in response to selection imposed by warmer climates, and does the degree of adaptation potential differ among traits and populations? In response to Q1, we hypothesized that genetic variance in quantitative traits would be greatest at the center of the species' range, where population connectivity and gene flow are estimated to be higher than in northern or southern populations (H1) (Ribicoff *et al*., 2025). In regard to Q2, we further hypothesized that selection on quantitative traits related to climate change response would be strongest in gardens located farthest away from each population's home latitude (H2). Finally, for Q3, we hypothesized central populations to have a high potential to adapt to warmer conditions, due to their high (expected) genetic variance for traits related to climate, relative to the northernmost and southernmost populations (H3a). Alternatively, for Q3, we hypothesized that the populations at range edges could have the highest potential to adapt to climate change because they are already under selection pressures associated with either the high rate of change (northern edge) or the increased intensity of warming and drought, exacerbating stress already present (southern edge) (H3b).

## Materials and Methods

### Reciprocal transplant experimental design

The Adaptation to Climate and Environment (ACE) experiment was designed to test for local adaptation by reciprocally transplanting *Q. macrocarpa* L. seedlings from known families across three garden sites spanning a latitudinal gradient, as described in Rea *et al*. (2024) (see Supporting Information Fig. S1). Acorns from Minnesota, Illinois, and Oklahoma populations were collected and germinated in 2018 and 2019 from individual mother trees. The seeds were germinated in a common environment in Vallonia, Indiana (see Fig. S2 for Vallonia climate data), then in spring of 2021 planted into common gardens in Minnesota (Cedar Creek Ecosystem Science Reserve, East Bethel, MN, USA), Illinois (Morton Arboretum, Lisle, IL, USA), and Oklahoma (Kessler Atmospheric and Ecological Field Station, Purcell, OK, USA). In each of the common gardens, we planted 10 individuals from 20 families, totaling 200 individuals from each of the three populations (Minnesota, Illinois, and Oklahoma) for 600 total trees in each garden. The bare‐root seedlings were planted 1 m apart in a randomized block design, with at least one individual from each family planted in each block, and provided supplemental irrigation during the growing season for 2 yr after planting. Climate data (Table 1) averaged for the years 2021–2023 was acquired from ERA5 (Copernicus Climate Change Service, 2023; Hersbach *et al*., 2023) using garden coordinates. The index of moisture (IM) was calculated as the difference between precipitation and potential evapotranspiration, where a lower IM indicates a greater water deficit and therefore higher aridity. Potential evapotranspiration was calculated using the Thornthwaite (1948) method. Nutrient differences in the gardens' soils were explored in Rea *et al*. (2024), and indicate that the Oklahoma and Illinois gardens' soils are richer in nitrogen and carbon than that of the Minnesota garden.

**Table 1:** ERA5 climate data for gardens averaged over the years 2021–2023, including mean annual temperature (MAT), mean annual precipitation (MAP), and the index of moisture (IM) (lower IM indicates higher aridity).

Garden	MAT (°C)	MAP (mm)	IM
OK	16.9	2.2	−107.2
IL	11.9	3.0	−91.0
MN	7.4	2.3	−77.4

### Spectral measurements

We took field measurements in 2023, at the peak of the growing season in each garden (OK: mid‐June, IL: early July, MN: late July). To reduce the effects of phenological changes in physiology, we attempted to measure gardens at a time when they had accumulated a similar number of growing degree days (*c*. 4000), which we approximated with the National Phenology Network's Data Visualizer (USA National Phenology Network, 2025). We took one leaf hyperspectral reflectance measurement per leaf on the uppermost, fully expanded, least damaged leaf on each tree canopy during peak light hours (between 10 am and 3 pm), using a portable field spectrometer (SVC HR‐1024i; Spectra Vista) and a leaf clip with an internal light source (LC‐RP PRO; Spectra Vista). Because the gardens include a randomized block design, our measurements were randomized across maternal families, which helped to minimize temporal effects on measurements. Spectral reflectance was measured to rapidly acquire vast amounts of phenotypic data nondestructively with a single measurement. The spectral reflectance encompassed the wavelength range 340–2500 nm in 1024 spectral bands. The breakpoint in the spectra due to different spectral sensors was matched by splicing the spectra at 990 nm and 1100 nm and interpolating 5 nm and 1 nm, respectively, using the match_sensors function in the spectrolab package v.0.0.18 (Meireles *et al*., 2021) in R v.4.3.0 (R Core Team, 2023).

### Spectral models to classify populations

We wanted to find wavelengths that were important in discerning populations. We used partial least squares discriminant analysis (PLSDA) models to predict population classification from the spectra in each garden in 2023 using methods adapted from Schweiger *et al*. (2018) and Sapes *et al*. (2022). We then resampled the spectra to every 10 nm between 400 and 2400 nm to reduce autocorrelation using the function resample() from spectrolab. We developed the PLSDA models in two stages. In the first stage, our goal was to ascertain the number of components needed for the final model, which we developed in the second stage. We divided the spectral data by garden and selected population as the class to be predicted by the model. For each set of garden data, we partitioned the spectral data using 80% of the data for training the model and 20% reserved for testing the accuracy of the model. We created the model using the train function in the caret package v.6.0‐94 (Kuhn *et al*., 2023) using the ‘simpls’ method, which is a simple PLS method. We used the ‘Bayes’ option as the probability method to account for prior probability distributions among classes, and we used a bootstrap resampling method. We evaluated the kappa values (a model performance statistic to compare model performance to random classification; Cohen, 1960) after 200 iterations of the model, and used a Tukey *post hoc* test and selected the number of components that corresponded to a maximal plateau in the kappa values, where there was no significant difference from increasing the number of components. To develop the final model, we created another data partition with 15 spectra selected from each population for the training set, and the rest of the spectral measurements were reserved for testing (see Table S1 for the total number of individuals used in the model). We then ran PLSDA models using the plsda() function from the caret package (Kuhn *et al*., 2023). We extracted the loadings from the PLSDA models and selected the reflectance for wavelength bands 700, 900, and 1400 nm, which had the highest loadings to use as traits in selection analysis and heritability estimates.

### Physiological traits predicted from spectra

#### Inversion models to predict traits from spectra

We predicted traits from the spectra using PROSPECT models in the R package prospect v.1.6.2 (Féret & De Boissieu, 2024). PROSPECT models are used to simulate the optical properties of a leaf based on trait data (Féret & De Boissieu, 2024). Here, we employ the inversion of PROSPECT using optical properties of leaves to simulate leaf traits (including Chl, carotenoids, anthocyanins, equivalent water thickness, leaf mass per area (LMA), proteins, and carbon‐based constituents) using the ANGERS experimental database used to calibrate the PROSPECT models. To prepare the spectral data for the model, we matched the sensors using the spectrolab function match_sensors, spliced at 990 and 1900 nm, and resampled the spectra to every 1 nm between 400 and 2450 nm using the spectrolab function resample (Meireles *et al*., 2021) to match the spectral intervals in the ANGERS database. We calculated the transmittance from our spectral data using the equation *T* = 1 − *R*, where *T* is transmittance and *R* is reflectance. We used the function FitSpectralData from the prospect package to adjust the spectral domain to fit the leaf optical properties. We then conducted the inversion with the function Invert_PROSPECT for the D version of prospect because it was able to model our traits of interest (LMA and anthocyanins) (Féret *et al*., 2019; Spafford *et al*., 2021).

#### Spectral indices

We calculated additional spectrally‐derived traits through the use of spectral indices. We calculated the Chlorophyll : Carotenoid Index (CCI) using the equation

(Eqn 1) $$\mathrm{CCI}=\frac{R531-R645}{R531+R645}\;\left(\text{Gamon}\;\mathrm{et}\;\mathrm{al}.,2016\right)$$

where R531 is the reflectance at 531 nm and R645 is the reflectance at 645 nm. The CCI has been positively correlated (*R* ^2^ = 0.47 for oaks; Wong *et al*., 2019) with photosynthesis rates, and closely mirrors shifts in measured Chl : carotenoid pigment ratios; that is, a larger CCI is indicative of a greater ratio of Chl to carotenoids in the leaf (Gamon *et al*., 2016). This index is also useful to compare across gardens as it is not particularly sensitive to diurnal changes in xanthophyll pigments, which would affect the interpretation of carotenoid levels (Gamon *et al*., 2016). We also measured the water band index (WBI) using the equation

(Eqn 2) $$\mathrm{WBI}=\frac{R970}{R900}\;\left(\text{Penuelas}\;\mathrm{et}\;\mathrm{al}.,1997\right)$$

where R970 is the reflectance at 970 nm and R900 is the reflectance at 900 nm, to estimate plant water concentration (positively correlated *R* ^2^ = 0.66 across species; Penuelas *et al*., 1997). Increases in WBI indicate higher plant, or in our case – leaf, water concentration, meaning more water per unit of dry mass.

### Morphological measurements

We measured initial height and basal diameter of each individual when it was planted in the gardens, and then remeasured height and basal diameter at the end of each growing season for 2 yr. We calculated the conoid stem volume as a combined estimate of plant size as follows:

(Eqn 3) $$V=H\times \pi \times {D_{\mathrm{rc}}}^{2}/12$$

where *V* is the tree volume, *H* is the height of the tree from the root collar to the apical meristem, and *D*_rc_ is the stem diameter at the root collar. Relative growth rate (RGR) was calculated as follows:

(Eqn 4) $$\mathrm{RGR}=\ln\left(V_{\text{final}}\right)-\ln\left(V_{\text{initial}}\right)/T_{\text{final}}-T_{\text{initial}}$$

where *V* is stem volume and *T* is time in total days. In addition, we measured leaf thickness with a digital caliper in one location in the middle of the leaf on the right side of the midvein to avoid any major veins.

### Traits used in analyses

We grouped traits into three categories: physiological, morphological, and spectral. For the physiological traits, we chose the WBI as a measure of plant water status, CCI as a measure of plant photosynthetic capacity, and anthocyanin as a measure of stressors including photoprotection, herbivory defense, heavy metals, and drought (Gould, 2004). Each of the physiological traits was chosen due to its expected importance for survival and function under future circumstances in climate change (increased drought and increased herbivory due to increased insect presence). There are caveats to directly interpreting spectral indices as traits, primarily that indices may be indicative of multiple physiological or structural functions; for example, CCI indicates photoprotective pigment pools as it is based on a ratio of Chl : carotenoid pigments (Gamon *et al*., 2016). However, CCI is primarily interpreted in the literature from the lens of investment in photosynthetic activity over photoprotection, and is the interpretation we use in this study (Gamon *et al*., 2016; Springer *et al*., 2017; Wong *et al*., 2020). We find spectral‐based traits useful in this study as nondestructive, high‐throughput measurements of physiological functions.

We chose RGR, LMA (derived from Prospect models), and leaf thickness as our morphology traits. We use the term morphology to group these traits as it pertains to the shape and size of various parts of the trees. The RGR of the stem was chosen for its connection to carbon sequestration and its importance for climate mitigation. LMA and leaf thickness were selected due to their expected associations with increased temperature (Groom *et al*., 2004; Wright *et al*., 2017).

For spectral traits, we selected three wavelengths with the top 10 highest loadings from the PLSDA model that also had known biological significance and used the reflectance at that wavelength as the trait value. We selected 700 nm (R700) as it is associated with the red edge, which has known associations with photosynthesis (Curran *et al*., 1990), 900 nm (R900) as it is associated with cellular structure (Knipling, 1970), and 1400 nm (R1400), which is associated with the spectral water absorption feature in the spectra (Pu *et al*., 2004; Mobasheri & Fatemi, 2013). These spectral traits encompass both physiology and morphology and allow us to test our ability to detect genetic variance and selection on traits using single wavelength bands.

### Quantitative genetic variation in populations

To address Q1, we partitioned the phenotypic variance in traits as

(Eqn 5) $$V_{p}=V_{g}+V_{e}=V_{a}+V_{d}+V_{i}+V_{m}+V_{\text{resid}}$$

where *V*_p_ is the phenotypic variance, *V*_g_ is the genetic variance, *V*_e_ is the environmental variance, *V*_a_ is the additive genetic variance, *V*_d_ is the genetic variance due to dominance, *V*_i_ is the genetic variance due to epistatic interaction, *V*_m_ is the environmental variance due to maternal effects, and *V*_resid_ is the residual variance. To partition the variance and obtain estimates of genetic variance (*V*_g_) and environmental variance (*V*_e_), we used the restricted maximum likelihood (REML) method (Shaw, 1987) executed in the QUERCUS program of Shaw & Shaw (1994). The QUERCUS program can be used to calculate components of genetic variance, including additive genetic, dominance, maternal, paternal, and residual variance. Using REML in these calculations as opposed to maximum likelihood (ML) eliminates bias due to unbalanced numbers of individuals within families (due to mortality in the case of this study) and loss of degrees of freedom for estimation of fixed effects (Shaw, 1987). The REML model eliminates the bias in ML by standardizing to a mean of zero to achieve estimates similar to ANOVA with the properties of ML, and allows estimates to be constrained to avoid negative values (Shaw, 1987). The calculations can be done using relationships between siblings, using the expectations of Mendelian inheritance (Falconer & Mackay, 1996 p. 171–172). We assumed that variances due to dominance (variance due to dominant gene action that masks recessive alleles) and maternal genetic effects (the contributions of the maternal environment to offspring provisioning) were zero; these components could not be estimated due to missing information on paternal individuals. Thus, we were solely estimating *V*_a_, and *V*_e_, and therefore for this manuscript refer to the genetic estimate of variance from QUERCUS as *V*_g_, to acknowledge the other variance partitions unaccounted for. We included the garden block as a fixed effect. We approximated the pedigree by using the known mother trees and assuming the families were full siblings and using 0 to indicate that the father is unknown. We ran the QUERCUS model to acquire variance estimates for each population in each garden. We calculated broad‐sense heritability (*H* ^2^) as

(Eqn 6) $$H^{2}=V_{g}/V_{P}$$

where *V*_P_ is the sum of the environmental (*V*_e_) and genetic variance (*V*_g_) (Falconer & Mackay, 1996 p. 172). We estimated the variance components assuming families were made up entirely of full siblings, then calculated *H* ^2^ for full siblings. Previous literature on the closely related species, *Quercus lobata* and *Q. alba* indicate that the number of pollen donors in a family averages between 3.7 and 8.2, with the number of donors decreasing with smaller stand sizes. Given that the populations are probably not made up of entirely full‐sibling groups, our estimates of genetic effects could be lower than if we had the full pedigree information (Austerlitz & Smouse, 2001; Sork *et al*., 2002). However, estimates may also be inflated due to the inability of the experimental design to separate maternal effects that could make siblings share higher phenotypic similarity (Mitchell‐Olds & Rutledge, 1986; Shaw *et al*., 1994). The inflation of estimates of genetic variance would still be present and exacerbated by assumptions of half‐siblings, thus we chose to use full‐sibling calculations since we lacked phenotypic data for the parents and pedigrees for the offspring. Furthermore, it is unknown whether bur oaks self‐pollinate, which can lead to further departures from the half‐sibling estimation (Gauzere *et al*., 2020). Using the full‐sibling calculation can help balance the aforementioned opposing biases that could inflate or deflate estimates.

We ran the QUERCUS models for sets of three traits at a time, corresponding to physiological traits inferred from spectra, spectral wavelengths, and growth/morphology traits, resulting in three models for each population in each garden, for a total of 27 models in this analysis (see Table S2 for the number of individuals included in models). RGR and LMA were multiplied by 1000 to increase the digits reported in the QUERCUS program (see Table S3 for a summary of traits used in the analysis and their biological interpretation).

We performed log‐likelihood ratio tests (LRTs) to compare the log likelihood of the models estimating *V*_g_ to models where *V*_g_ is constrained to zero using the equation

(Eqn 7) $$\mathrm{LRT}=-2\times \left(L_{0}-L_{\max}\right)$$

where *L*_0_ is the log likelihood of the null hypothesis model that excludes *V*_g_ and *L*_max_ is the full model. We compared the LRT to a chi‐square probability distribution at a significance level of 0.05 with 1 degree of freedom (corresponding to the single parameter that distinguishes the full model from the null model). The estimates were considered significant if the LRT was greater than the chi‐square probability. Standard errors were reported as the square root of the corresponding element of the asymptotic covariance matrix of the estimates.

### Fitness estimation

We estimated overall fitness for each individual in each garden using ML aster models (Geyer *et al*., 2007; Shaw *et al*., 2008). Aster models are an approach to life history analysis that produces a single combined estimate of lifetime fitness from multiple dependent fitness components. They resolve the issue of non‐independence of variables by modeling the fitness components expressed later as dependent on components expressed earlier in a plant's lifetime, and allow for different sampling distributions that properly fit each component (Shaw *et al*., 2008). The fitness components for our model were two‐year survival (binomial distribution) and height dependent on 2‐yr survival (normal distribution) (see Fig. S3 for height and mortality data). Height was chosen as the final predictor of fitness because size can be a predictor of eventual fecundity (Cornelius, 1994; Gamache & Payette, 2004). Differences in height and mortality across gardens for each population are shown in Fig. S3, demonstrating the greatest height in the Illinois garden, as well as the highest mortality in the Oklahoma garden. The Oklahoma population tends to be taller than the other populations. These population × environment differences are explored in Rea *et al*. (2024).

We used separate models for each garden to acquire estimates of fitness for each individual (see Table S2 for number of individuals included in models). The predictors included in the models were:

(Eqn 8) F=M+S+B

where F is predicted fitness, and maternal families (M), height at initial planting (S), and garden block (B) are treated as fixed effects. While maternal family could be considered a random effect, we chose to keep it as a fixed effect to simplify modeling, given that inclusion of random effects drastically increases computational challenges to aster models. We included height at initial planting to account for potential maternal effects that influenced early fitness. In the Illinois garden, the model was parameterized without block as an additional fixed effect as block was determined not to be significant in that instance through the use of omnibus tests. We used the predict function to estimate fitness from the model.

### Selection on traits across gardens

To address Q2, we examined phenotypic selection on traits at the garden‐specific and population‐specific level using multiple regression following Lande & Arnold (1983). First, we calculated relative fitness for each individual by dividing the individual's fitness by the mean fitness for the garden. Because we included survival as a part of our aster fitness estimation, survival is included as a part of the selection values calculated from these fitness estimates, and differences in mortality are accounted for across gardens (see Fig. S3 for mortality rates). To avoid multicollinearity, we regressed the phenotypes used for quantitative genetic estimates with height (the predictor of fitness in our aster models) to determine the correlation between these traits and height, and used this regression to examine the variance inflation factors (VIFs) with the vif() function in the car package v.3.1‐3 (Fox *et al*., 2023). We eliminated traits with VIF scores greater than five, resulting in anthocyanins (ANT), the carotenoid : Chl index (CCI), the WBI, spectral reflectance at 700 nm, leaf thickness, and the relative volume growth rate (RGR_Vol) to be used in the selection analyses (see Fig. S4 for correlations among traits). These traits were standardized to have a mean of zero with a SD of one (see Table S2 for number of individuals included in models).

For the garden‐specific analysis, we used a separate linear mixed model for each garden regressing relative fitness against the four traits, with block and population as random effects (M1a, see Table S4 for mathematical equations for models used in this analysis). The partial regression coefficients of the fixed effects in the models were extracted as the linear selection gradients, a measure of direct selection on the phenotype (β values), which tell us about the direction and strength of selection. We also calculated the selection differential (S), which here we interpret as a combined measure of direct selection on a given trait, mediated by its correlations with other traits leading to indirect selection. We calculated the selection differential as the covariance of relative fitness with the vector of traits.

To evaluate whether selection was stabilizing (i.e. intermediate phenotypes favored), we conducted quadratic regressions of relative fitness on the traits (M2a), separate from the linear regressions used in the analysis of direct selection. From the quadratic regression, we extracted the partial regression coefficients corresponding to γ values, which predict the curvature of the selection surface and were interpreted following Kingsolver *et al*. (2001) to indicate stabilizing selection if the gradient is negative and disruptive selection if the gradient is positive. We evaluated the significance of the model coefficients β and γ for significance using a two‐tailed Student's *t*‐test.

For the population‐specific analysis, we subset the data by garden and population and conducted the same analysis (M1b and M2b) to acquire β, S, and γ values for each population in each garden. The non‐linear selection gradients were visualized with the R function persp() to make three‐dimensional graphs with relative fitness and combinations of two trait values at a time. The surfaces were plotted as a mesh surface using the loess() function to estimate the surface from the trait values. This visualization allows us to assess the combined effects of traits on fitness and observe peaks at intermediate values corresponding to stabilizing selection.

### Response to selection

To address our final question (Q3), we evaluated the response to the selection of each population in each garden using the equation:

(Eqn 9) $$R=H^{2}\times S$$

where *R* is the response to selection, *S* is the selection differential calculated as the covariance of relative fitness with the vector of traits, and *H* ^2^ is broad‐sense heritability. This calculation is based on the Breeder's equation, which predicts the response to selection as the change in mean of a population expected from one complete generation to the next (Lush, 1937). The Breeder's equation uses narrow‐sense heritability calculated from additive genetic variance. Here, we used broad‐sense heritability because we did not have controlled crosses and could not eliminate transgenerational and other genetic effects. By using broad‐sense heritability instead of narrow‐sense heritability in this equation, we are including multiple components of genetic variance, including additive, dominance, and epistatic variance. Because broad‐sense heritability includes genetic components of dominance and epistatic variance, the phenotypes are not necessarily inherited across generations. As such, the response to selection (*R*) in this study suggests how much response to selection could be expected in the short term with the current genetic architecture of the population(s). Larger values of *R* indicate greater change in mean trait value, and the sign of *R* matches the sign of the selection differential, indicating the amount the mean trait value is expected to increase (positive *R*) or decrease (negative *R*).

## Results

### Quantitative genetic variation

**Fig. 1:**
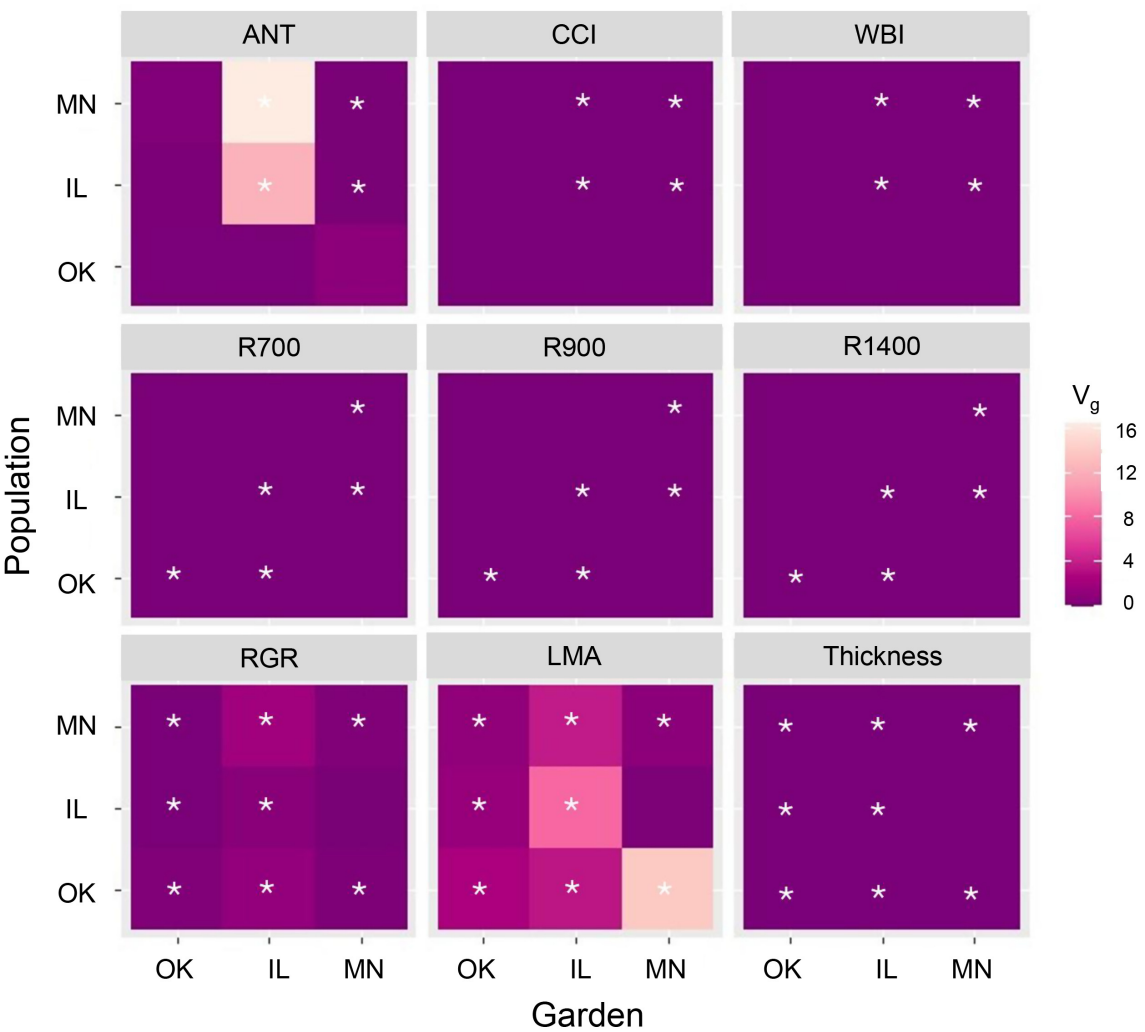
Heat maps indicating the magnitude of genetic variance (*V*_g_) for selected traits in each *Quercus macrocarpa* L. population for each garden. Traits were grouped for QUERCUS analysis into *physiological traits* (anthocyanins (ANT), Chl : carotenoid index (CCI)), *spectral reflectance bands* at 700, 900, and 1400 nm, and *morphological traits* (leaf mass per area (LMA), relative growth rate (RGR), and leaf thickness), and water band index (WBI). Significance at the *P* < 0.05 level was calculated with likelihood ratio tests and is indicated by white asterisks in the cells.

We found significant genetic variance (*V*_g_) for many of our traits across populations and gardens (Fig. 1). We report SE and variance due to environment in the supplement (Table S5). For the physiological traits, the Minnesota and Illinois populations both expressed significant *V*_g_ in the Minnesota and Illinois gardens, whereas *V*_g_ was not significant for the physiological set of traits (WBI, anthocyanin, or CCI) in the Oklahoma garden for any population. The *V*_g_ for anthocyanin (ANT) was highest for both the Illinois and the Minnesota populations in the Illinois garden. We did not find significant *V*_g_ for the Oklahoma population in any garden for the physiological set of traits.

For the spectral analysis (R700, R900, R1400), we found significant *V*_g_ for all populations in their home gardens. We did not detect significant *V*_g_ expressed for spectral bands in the Minnesota population outside of its home garden, nor for the Illinois population in the Oklahoma garden. The *V*_g_ was highest for R1400 and R900 for the Oklahoma population in its home garden and was the highest overall for all populations.

The Minnesota and Oklahoma populations expressed the highest *V*_g_ for RGR in the Minnesota garden. The *V*_g_ of RGR was lowest in the Illinois population; however, the *V*_g_ of RGR for the Illinois population was greater in the home garden than in the Oklahoma garden, suggesting that conditions in the Oklahoma garden impact genetic variance. Our ability to detect genetic variance in the Oklahoma garden is also likely limited by the high mortality in the Oklahoma garden. The Oklahoma population expressed high *V*_g_ for LMA in the Minnesota garden. By contrast, *V*_g_ of leaf thickness was low for all populations in all gardens. For growth and morphology traits, *V*_g_ was generally significantly greater than 0, except for the Illinois population in Minnesota.

**Fig. 2:**
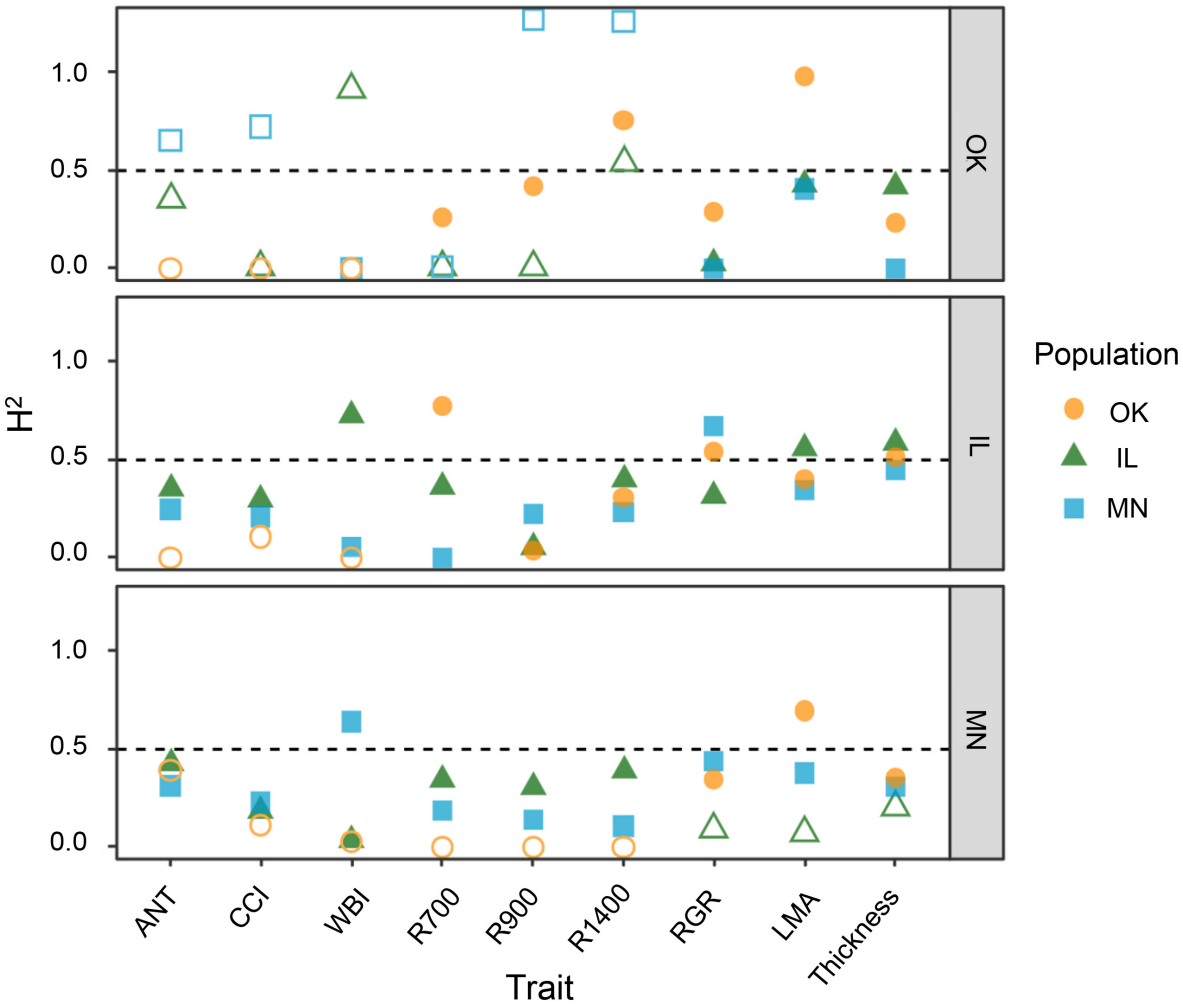
Broad‐sense heritability (*H* ^2^) for anthocyanin (ANT), Chl : carotenoid index (CCI), water band index (WBI), reflectance at 700, 900, and 1400 nm (R700, R900, and R1400), relative growth rate (RGR), leaf mass per area (LMA), and leaf thickness in each of the three *Quercus macrocarpa* L. populations across the three gardens. Each panel corresponds to a garden, with orange circles indicating the value in the Oklahoma population, green triangles representing the Illinois population, and blue squares representing the Minnesota population. Filled shapes indicate that genetic variance was significant, determined using likelihood ratio tests. Dashed horizontal lines at *H* ^2^ = 0.5 indicate the point above which genetic variation contributes more than environmental variation to among family differences.

Heritability estimates (*H* ^2^) of full siblings ranged from 0 to 0.98; heritabilities for morphology and growth traits were generally higher than for the other trait groupings (physiological and spectral) (Fig. 2). Of the heritability estimates that were significantly different from 0, some were greater than 0.5, indicating that differences in traits among families have a strong genetic basis. Instances of *H* ^2^ > 0.5 were generally associated with morphological traits, but did not follow a discernible pattern across populations or gardens. All of the populations expressed greater heritability of RGR and leaf thickness in the Illinois garden (Fig. 2, panel IL) and lowest in the Oklahoma garden (Fig. 2, panel OK). For the Minnesota and Illinois populations, each had substantially higher heritability of WBI when in their home garden (Fig. 2, panels MN and IL). The Oklahoma population had higher heritabilities of 900 nm reflectance band and the 1400 nm reflectance band in its home garden (Fig. 2, panel OK), but lower heritability of the 700 nm band at home compared to in the Illinois garden (Fig. 2, panel IL).

### Directional selection analysis

#### Garden level

We found significant directional selection (β) in each of the gardens for different phenotypes (Table 2). The β of WBI was negative in each of the gardens, indicating selection for lower WBI, though it was only significant in Minnesota. RGR had the greatest selection strength and a positive β value, indicating strong selection for fast growth rates in the Illinois garden. In the Illinois garden, β for reflectance at 700 nm was negative, indicating significant (*P* < 0.05) selection for lower values of reflectance at 700 nm and greater RGR. In the Oklahoma garden, there was a strong, positive, significant (*P* < 0.05), selection for LMA, indicating selection for thicker leaves. The selection differential (*S*) was in the same direction as the significant β values (WBI, R700, LMA, and RGR). *S* was also similar in magnitude to the significant β values, further supporting the expected directional selection on these traits.

**Table 2:** Garden‐level selection analysis selection coefficients (differentials and linear and quadratic gradients) for populations of *Quercus macrocarpa* L.

Trait	Selection parameter	Garden		
		MN	IL	OK
Anthocyanins	β	−0.0396 ± 0.03	0.0253 ± 0.01 +	−0.0411 ± 0.04
Chlorophyll : Carotenoid Index	β	−0.0079 ± 0.02	0.0075 ± 0.01	−0.0071 ± 0.05
Water band index	β	−0.0333 ± 0.02*	−0.0087 ± 0.02	−0.0026 ± 0.06
Reflectance at 700 nm	β	0.0181 ± 0.03	−0.0364 ± 0.02*	0.0333 ± 0.04
Relative growth rate	β	0.0136 ± 0.01	0.0755 ± 0.02**	−0.0150 ± 0.04
Leaf mass per area	β	0.018 ± 0.03	0.0232 ± 0.02	0.123 ± 0.05*
Leaf thickness	β	−0.0075 ± 0.02	0.0076 ± 0.02	0.0213 ± 0.05
Anthocyanins	*S*	−0.0247	0.0318	−0.0344
Chlorophyll : Carotenoid Index	*S*	−0.0335	0.0403	0.1
Water band index	*S*	−0.0376	−0.0403	−0.1415
Reflectance at 700 nm	*S*	0.0466	−0.0517	0.0905
Relative growth rate	*S*	0.0123	0.1397	0.1194
Leaf mass per area	*S*	−0.0019	0.0682	0.1603
Leaf thickness	*S*	−0.0036	0.0384	0.1353
Anthocyanins	γ	0.0168 ± 0.02	0.0081 ± 0.002**	0.0053 ± 0.01
Chlorophyll : Carotenoid Index	γ	−0.0076 ± 0.007	−0.0002 ± 0.008	0.0204 ± 0.02
Water band index	γ	−0.0099 ± 0.01	−0.0193 ± 0.008*	−0.0224 ± 0.03
Reflectance at 700 nm	γ	0.02 ± 0.01+	−0.0167 ± 0.007*	0.0044 ± 0.02
Relative growth rate	γ	−0.0045 ± 0.006	−0.0105 ± 0.009	−0.0078 ± 0.02
Leaf mass per area	γ	−0.021 ± 0.02	0.008 ± 0.008	0.0175 ± 0.03
Leaf thickness	γ	−0.0028 ± 0.009	−0.0073 ± 0.008	0.0127 ± 0.02

β values indicate directional selection, with the magnitude indicating strength of selection and the sign indicating the direction. *S* indicates the selection differential and is interpreted similarly to β. γ indicates quadratic selection gradients. Standard errors are given next to the coefficients/differentials. Significance levels are given by: **, *P* < 0.01; *, *P* < 0.05, and + indicates *P* < 0.1, calculated using a two‐tailed Student's *t*‐test.

#### Population × garden level

**Fig. 3:**
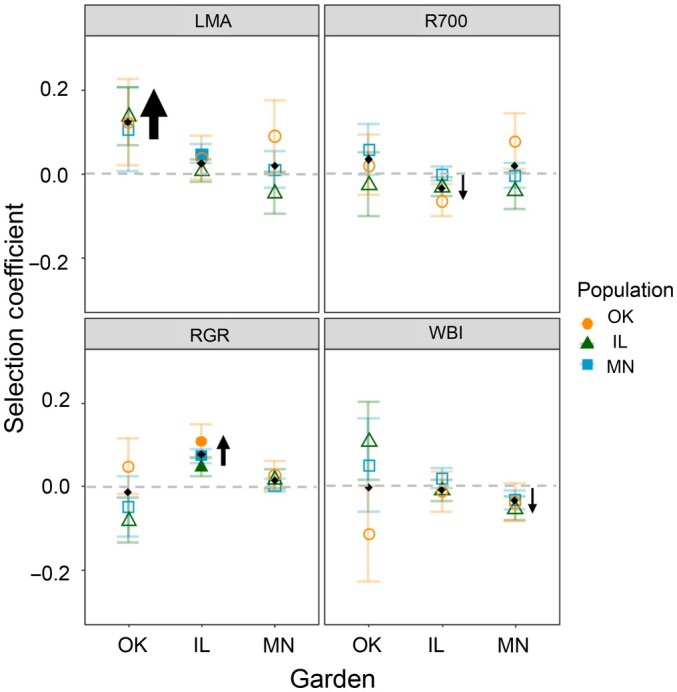
Comparing directional selection in each garden and population. Each panel corresponds to a selection in the traits: leaf mass per area (LMA), relative growth rate (RGR_Vol), reflectance at 700 nm (R700), and the water band index (WBI). The black diamond is the selection gradient from the garden‐wide analysis, and orange circles represent the *Quercus macrocarpa* L. population by garden selection analysis gradient of the Oklahoma population; green triangles represent the Illinois population, and blue squares represent the Minnesota population. Filled shapes indicate selection gradients with *P* < 0.05 at the population level, determined using a two‐tailed Student's *t*‐test. Black arrows point in the direction of the significant garden‐wide selection, and the size of the arrow indicates the strength of selection. Error bars indicate SE. A gray dashed line denotes the 0 mark for selection gradients.

**Fig. 4:**
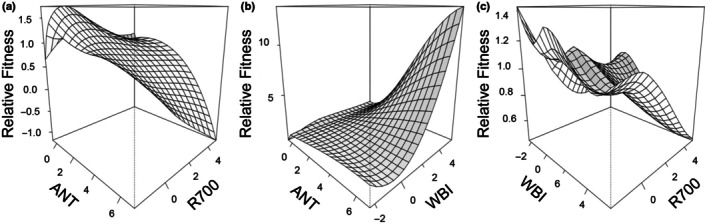
Three‐dimensional models of quadratic selection on traits of *Quercus macrocarpa* L. in the Illinois garden. Relative fitness (unitless ratio of individual aster fitness divided by the overall mean fitness for each garden) is plotted on the y axis against combinations of standardized traits: Anthocyanin × reflectance at 700 nm (R700) (a), Anthocyanin × water band index (WBI) (b), and WBI × R700 (c) to examine selection surfaces for curvature indicating stabilizing selection. Two‐dimensional plots of selection and relative fitness quadratic selection lines included in Supporting Information Fig. S5.

We analyzed directional selection at the population level to determine whether the direction and strength of selection were similar for foreign populations to the local population, particularly in warmer gardens (Fig. 3). In the Illinois garden, four traits had significant directional selection (LMA, WBI, R700, and RGR), and selection was in the same direction for all populations for these traits. The strength of selection was stronger on the Oklahoma population for both R700 (Fig. 3, panel R700) and the RGR (Fig. 3, panel RGR_Vol) than on the other populations, though the selection was not significant for any one population individually. In the Oklahoma garden, selection for LMA was in the same direction for all populations, and selection strength was similar (Fig. 4, panel LMA). However, the RGR was in the opposite direction for the local population compared to the overall selection direction, but nonsignificantly. For WBI, we saw the opposite pattern, with Illinois and Minnesota populations in the opposite direction of the overall direction of selection, but the local population in the same direction (Fig. 4, panel WBI).

### Quadratic selection analysis

The gradients for quadratic selection analysis were significant (*P* < 0.05) in only the Illinois garden for anthocyanins, WBI, and R700. The WBI and R700 quadratic selection gradients were negative, indicating stabilizing selection (Table 2). However, the anthocyanin selection gradient was positive, indicating disruptive selection. The curvature of the selection surfaces peaked at intermediate values for R700 with anthocyanins (Fig. 4a) and with WBI (Fig. 4c), consistent with stabilizing selection for R700. The surfaces for WBI and anthocyanins were relatively flat (Fig. 4b).

### Breeder's equation to predict response to selection (R)

**Fig. 5:**
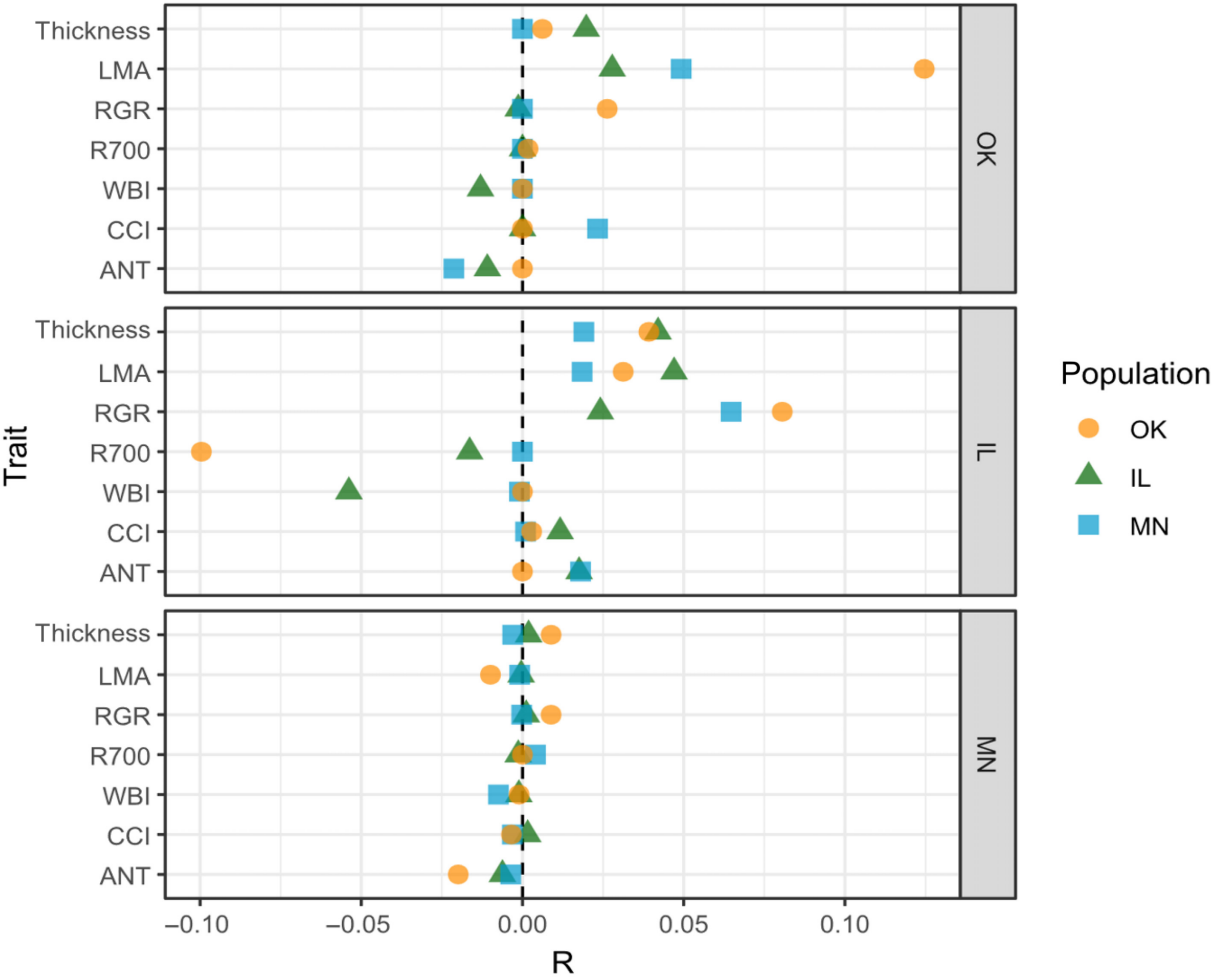
Breeder's equation values (*R*) used to predict evolutionary change for *Quercus macrocarpa* L. in anthocyanin (ANT), Chl : carotenoid index (CCI), water band index (WBI), reflectance at 700 nm (R700), relative growth rate (RGR), leaf mass per area (LMA), and leaf thickness due to selection. Each panel corresponds to a garden, with orange circles indicating the value in the Oklahoma population, green triangles representing the Illinois population, and blue squares representing the Minnesota population. A vertical dashed line denotes response to selection (*R*) of 0 to better distinguish positive or negative selection responses.

We found high selection responses in the Oklahoma population for increasing LMA in the Oklahoma garden and increased RGR in the Illinois garden (Fig. 5, panels OK and IL). We found similar trends in the selection response of LMA and RGR for the Minnesota population, though to a lesser degree than in the Oklahoma population. We found high expected decreases in R700 mean for the Oklahoma population in the Illinois garden, and high expected decrease in WBI mean for the Illinois population in the Illinois garden (Fig. 5, panel IL). The Illinois population always had a greater response to selection in its home garden compared to other gardens, whereas the Minnesota population exhibited low response to selection in its home garden (Fig. 5, panel MN).

## Discussion

Using a reciprocal transplant experiment, we tested the potential of three populations of bur oak (*Q. macrocarpa*) across a latitudinal gradient to adapt to different environmental and climatic conditions. We found evidence for high broad‐sense heritabilities, particularly in morphological leaf traits and growth rates (Fig. 2). Our findings align with expectations from prior studies showing high inherited responses in morphological leaf traits (Everingham *et al*., 2024). We found varying strengths and directions of selection across gardens, with RGR and LMA were under strong positive directional selection (Fig. 3) in both the Illinois and Oklahoma gardens. This pattern of strong directional selection indicates that families with higher LMA and higher RGRs have higher predicted fitness. We found high heritability estimates of RGR for all three populations in the Illinois garden, indicating that adaptation potential for RGR was high where growth conditions appear to be optimal for all populations (Rea *et al*., 2024). Estimated heritability was also high for LMA, particularly for the Oklahoma population in the Oklahoma garden. Consequently, the Oklahoma population expressed a high response to selection (*R*) for LMA in Oklahoma (Fig. 5). This result indicates that with the existing genetic architecture of this population, high heritability and strong selection could enable adaptation of LMA to increasingly stressful conditions imposed by the warmer and drier climate in Oklahoma.

The response to selection for populations outside their home garden can indicate how populations might adapt to future climate conditions. For example, the Minnesota population also had a high response to selection in the Oklahoma garden. Given that the Minnesota population had a lower heritability (*H* ^2^) estimate for LMA (<0.5), differences in the strength of selection among garden environments may have a greater impact on the population's response to selection than genetic variation. Adaptive responses to selection in traits that enhance fitness in warmer climates are especially important for the Minnesota population, which is expected to experience the greatest change in climate (Lee *et al*., 2021). Intense selection pressure from warmer climates may lead to high mortality rates in the Minnesota population as individuals lacking adaptations for warmer climates will likely die (Rea *et al*., 2024), however, the population may be able to adapt traits like LMA that would be adaptive in warmer climate conditions with the current genetic architecture present in the population.

### Differential selection gradients across gardens

The direction and strength of selection differed among gardens. RGR and LMA had the highest directional selection in all gardens. Strong selection for greater LMA in the Oklahoma and Illinois gardens, which aligns with findings in *Quercus suber* in Mediterranean ecosystems (Ghouil *et al*., 2020), would likely be beneficial in projected future climates because thicker leaves that have reduced surface area and increased water use efficiency are favored in hotter, drier climates in plants with long leaf lifespans (Wright *et al*., 2002, 2004). The selection for higher LMA in Oklahoma populations may be capturing increased cell density, which has been shown to increase in hot, dry summer climates and was highly heritable (*h* ^2^ < 0.5) in *Populus tridentata* (Dunlap & Stettler, 2001). Measurements of leaf lobedness in *Q. macrocarpa* wild populations and the ACE experiment indicate countergradient selection, where the southern populations have more leaf lobing, which may decrease the boundary layer and lower leaf water content, but in southern gardens, leaf lobing is low across all populations (Desmond *et al*., 2021; Ostrowsky *et al*., 2026). Leaves with high cell density may counterbalance the increased lobing to increase the boundary layer in the Oklahoma population (Givnish, 1979, 1987; Schuepp, 1993). Our findings indicate selection for thicker leaves in hotter southern climates to improve water use efficiency and leaf lifespans. Selection for higher RGR in the Illinois garden was likely due to lower stress in the Illinois garden that enabled higher growth rates, selecting for genotypes that could grow fastest in the absence of significant abiotic stress (Grime, 2006; Rea *et al*., 2024), and aligns with previous findings in western whitebark pine species that populations had greater growth and higher fitness in milder climates (Warwell & Shaw, 2017).

We found significant stabilizing selection, but only in the Illinois garden, for the reflectance at 700 nm (R700) and the WBI (Table 2), which may indicate that less stressful environments select for intermediate trait values. Traits related to productivity, such as photosynthetic activity (represented by R700 here), are expected to converge toward an optimum at the local scale (Grime, 2006). On the other hand, a positive coefficient of the quadratic selection gradient for anthocyanins indicated disruptive selection, suggesting that the less stressful environment allows for more variable and disparate trait values. However, it is possible that due to the stable environment in Illinois, tree(s) in that garden grew larger, increasing light variability in the canopy, which could have resulted in greater leaf variation in attributes such as anthocyanin levels as a consequence of exposure to differing light levels (Gould *et al*., 2000). The significant quadratic selection gradients were low in magnitude, with barely discernible peaks or troughs, consistent with the majority of findings for stabilizing or disruptive selection as reviewed by Kingsolver *et al*. (2001). It is possible that stronger stabilizing selection for the traits in our study could have been obscured by phenotypic covariances that were not measured in this study, leading to underestimation of stabilizing selection in the gardens (Travis, 1989).

Selection on WBI was in the same direction in all gardens, but was only significant in Minnesota. This latter finding is somewhat surprising, given the importance of water for plant functions; however, it should be noted that over the two most recent years of the experiment, chronic continental‐scale drought occurred and was especially severe in Minnesota. As a consequence of the drought, leaf water content was generally low, and only individuals that were able to maintain function with low leaf water content survived. As a result, we were only able to measure leaves from the surviving trees. It is thus possible that our finding of significant selection on WBI in the Minnesota garden could be a consequence of selection on unmeasured correlated traits that impacted survival during the drought, preventing us from adequately testing for independent effects of leaf water content on fitness. Nevertheless, the ability to maintain function and survive with low leaf water content is likely adaptive in drought. *Q. macrocarpa*, like other oaks (Cavender‐Bares & Bazzaz, 2000), tends to use an anisohydric (‘water spending’) strategy in response to drought, maintaining stomatal opening such that photosynthesis continues even during periods of water stress. The trees may increase foliar osmolyte concentration to maintain a water potential gradient enabling them to continue drawing water from the soil during drought (Chabot & Hicks, 1982; Kozlowski & Pallardy, 2002; McDowell *et al*., 2008; Kaproth *et al*., 2023). Due to the shorter growing season in Minnesota, this anisohydric strategy may be particularly important to achieve sufficient carbon gain for growth and overwintering respiration. On the other hand, selection on WBI, while not significant, in the Oklahoma garden is positive for both the Minnesota and Illinois populations and negative for the Oklahoma population, which suggests that greater leaf water content in trees from northern populations survive better in the hotter climate as it allows them to employ the anisohydric ‘water‐spending’ strategy. These results indicate that selection pressures differ on northern populations compared to the southern population in the warmest garden, which may have implications for how the populations respond to climate change. With increasingly arid conditions expected with climate change, the northern populations ‘spending on water may eventually put them at risk of desiccation and death.

We also found significant negative selection for R700 in the Illinois garden, indicating selection for reduced Chl content per unit leaf area. However, total leaf size was greater for all populations in the Illinois garden (Ostrowsky *et al*., 2026), suggesting that total Chl per leaf and overall light harvesting capacity were not diminished in these individuals, as evidenced by their much higher growth and photosynthetic rates in the Illinois garden (Rea *et al*., 2024). This selection for lower Chl content per leaf area may be the result of selecting for larger leaves to maximize carbon assimilation and enhance light capture across the entire leaf area.

### Population by garden selection analysis

Despite strong selection on LMA in the Oklahoma garden when populations are pooled, when analyzed individually, we do not detect significant selection for LMA at the population level in the Oklahoma garden. This is likely due to reduced prediction power due to mortality in the Oklahoma garden and may also be a result of historic selection limiting the amount of genetic variation existing in the populations. However, the direction of selection on northern populations is in the same direction as the local population and is in accordance with the whole garden direction of selection. In the least stressful (Illinois garden), we found significant positive selection for all populations, matching the direction of the overall garden selection. This finding further emphasizes the importance of the stable, low‐stress environmental conditions found in the Illinois garden for *Q. macrocarpa* growth.

### Quantitative genetic variance

Quantitative genetic variance was distributed heterogeneously among traits (Fig. 1). In general, morphological and growth traits had higher genetic variance across all populations compared to physiological and spectral traits. The visible range of the spectrum, as we see from the high genetic variance of anthocyanins (Fig. 1), as well as the heritability for R700 (Fig. 2) of the Oklahoma population in Illinois, is a highly heritable region of the spectrum, in agreement with Ballesta *et al*. (2022). Anthocyanins estimated by anthocyanin spectral reflectance index were also shown to be highly heritable in *Eucalyptus* spp. by Ballesta *et al*. (2022), which indicates that we can track genetic contributions of traits derived from spectral reflectance. We also found low, but significant heritability for CCI in the Illinois and Minnesota gardens, consistent with (Corbin *et al*., 2025), who found significant low heritability of carotenoids detected with a spectral index in *Poplar fremontii* transferred to cold to mid‐temperature climates. In addition, we find high heritability in the shortwave infrared region (R1400) of the spectra, where water absorption features are located, as well as suggesting that leaf water content may also have some genetic influence. While these results are promising for high‐throughput, nondestructive detection of heritability in traits, multiple factors can influence spectral responses to climate (Stefanski *et al*., 2025). As such, detection of heritability of certain indices or spectral bands may not indicate heritability of specific traits, but rather regions of the spectrum that may be heritable and useful to track in ongoing efforts to enhance adaptive capacity in populations in the face of climate change. It is important to note that the degree of genetic variance in a population tends to vary in different gardens and does not always translate into high heritability (as is the case for anthocyanins in our study). Low heritability despite high genetic variance could be a consequence of high environmental variance. We also note that the heritabilities were calculated assuming full‐sibling groups, providing a conservative estimate of heritability. Additional analysis with full pedigree information could reveal higher heritabilities than we report here.

We expected to find greater genetic variance in the Illinois population, given that it represents the center of the range, following the central‐marginal and latitudinal hypotheses reviewed by Guo (2012) and, specifically, findings from a similar experimental design by Etterson (2004). However, we found that the genetic variance for the Illinois population was not highest in every garden, but that all populations, particularly for morphological traits, had the highest genetic variance in the Illinois garden. The high genetic variance for morphological traits in the Illinois garden suggests that the conditions in that garden may lower selection pressure and allow for more genetic variance to flourish within the populations. Alternatively, high genetic variance could be a consequence of epigenetic effects that result in differences in gene expression (Yakovlev *et al*. 2012; Gugger *et al*. 2016). The findings that *Q. macrocarpa* deviates from the central‐marginal theory (genetic variance decreasing toward center of range)—along with 35.8% of species studied, reviewed by Eckert *et al*. (2008)—may have implications for their ability to expand their range in the face of climate change. Such deviations from the predictions of the central‐marginal hypothesis have evidence for potential range expansion from the edges of species ranges (Singhal *et al*., 2022).

Mortality reduced the number of individuals available to measure, decreasing statistical power to detect genetic variance, which particularly affected populations in the Oklahoma garden. Consequently, we did not find significant genetic variance for all populations in all gardens, likely due to sample size limitations. Our experimental design also may lead to some over‐estimation of genetic variance because we were unable to control for maternal effects and dominance effects due to the long life‐cycles of oak trees, which limited our ability to create crosses. The maternal environment can influence offspring trait expression and can lead to adaptive maternal effects that enhance offspring fitness (Mousseau & Fox, 1998). These adaptive maternal effects can be important to consider in moving genetic material (i.e. pollen or seeds) between environments, as seeds that are dispersed outside their maternal environment may not be adapted to their new environment and may have reduced fitness for a generation, but may recover fitness in the next generation of locally produced offspring in the new environment (Galloway & Etterson, 2007).

### Implications for northern populations with future climate change

If populations are to adapt rapidly enough to keep pace with the current rate of climate change, it is important that the response to selection of a population is of sufficient magnitude to enable rapid evolution. Negative genetic correlations among traits under selection can have the consequence of constraining the rate of adaptation to climate change (Etterson & Shaw, 2001). Based on models of climate change in Minnesota through 2069, climate is anticipated to increase temperatures by 3°C under the Coupled Model Intercomparison Project phase 3 (CMIP3) A2 (upper mid‐range) emissions scenario, bringing the mean annual temperature in Minnesota close to the current climate regime in Illinois (Galatowitsch *et al*., 2009). In our study, the Minnesota population demonstrated some degree of response to selection (R) with a change in mean trait value greater than 0 for many traits in the Illinois garden, including LMA, leaf thickness, anthocyanins, and particularly for relative growth rate, which is under significant directional selection in the Illinois garden. The high adaptation potential for the Minnesota population in the Illinois garden suggests that the Minnesota population will likely be able to adapt to the degree of climate change likely to occur in its home habitat in the short term (*c*. 30 yr). However, the long generation times of trees mean that it may take longer than 30 yr to produce offspring adapted to warmer climate conditions, leading to adaptational lags (Fastovich *et al*., 2025). Furthermore, the broad‐sense heritability calculation used to estimate response to selection includes genetic variance components (e.g. dominance effects) that are not necessarily inherited across generations. Consequently, over the long term (> 30 yr), the introduction of novel adaptive genes through gene flow may be an important factor in the degree to which Minnesota populations adapt to climate change, beyond what is possible through selection on current genotypes.

The potential for gene flow is higher between the Minnesota and Illinois populations because they are closer to each other than to the Oklahoma population and may be more genetically differentiated from the southern populations due to historical population structure (Ribicoff *et al*., 2025). An important means to increase forest resilience to long‐term climate change is to increase genetic diversity (Butler *et al*., 2012), which could be accomplished through the practice of population‐assisted migration or seed transfer of the southern populations and may be critical to alleviate negative climatic effects of maladapted populations (Browne *et al*., 2019). Gradual latitudinal transfers of < 1°C in mean annual temperature are likely to be low risk, based on adaptation potential for our Oklahoma population in Illinois. This finding is in agreement with work in the European oak *Quercus robur*, which shows evidence of local adaptation in growth (George *et al*., 2020). Such gradual transfers, along with directional selection through natural approaches or selective breeding, can enable adaptation of oaks to warmer and drier climates.

### Conclusion

Our results indicate that populations of bur oak at range edges have the potential to adapt to climate change. By contrast, we found that the stable climate and nutrient‐rich soils of the garden in the middle of our latitudinal gradient likely released trees from abiotic stresses found at the other ends of the gradient, leading to stabilizing selection and high growth rates. Collectively, our findings can inform strategies to select seed sources for assisted migration given expected climate change by providing insight into populations with the potential to adapt to hotter conditions. These results can also be used to target areas similar in climate and nutrient conditions for conservation that provide regions for populations to thrive.

Future studies could expand on the traits in this study to investigate selection on bud‐burst across populations and environments and evaluate genetic control on phenological cues, which could impact growth and survival. Including additional traits related to drought tolerance and resistance such as stomatal density, osmotic potential, and hydraulic conductivity could elucidate responses to selection pressures likely to be imposed by climate change.

## Competing interests

None declared.

## Author contributions

All authors contributed intellectually to the study. JCB designed the ACE experiment and managed its implementation. JCB, ALH and HRM managed the collaboration and secured funding for the study. LMSR led data collection, analysis and interpretation, and writing of the manuscript. JCB led nursery planting. CL, LW and MG led planting efforts in the gardens. LO, RAM, MG, LW and CL collected data. JCB, ALH and HRM edited early versions of the manuscript. All authors provided critical revision of the article and provided final approval of the version to be published.

## Disclaimer

The New Phytologist Foundation remains neutral with regard to jurisdictional claims in maps and in any institutional affiliations.

## Supporting information


**Fig. S1** Map of collections for each population, overlayed over species distribution for *Quercus macrocarpa* from United States Geological Survey.
**Fig. S2** Temperature and precipitation in Vallonia, Indiana Department of Natural Resources Nursery, where trees were germinated and grew from 2018 to 2021.
**Fig. S3** Box plot of heights for each population in each garden in 2023 and Percent mortality in 2023 in each population in each garden.
**Fig. S4** Correlation matrix of traits used in selection analysis.
**Fig. S5** Two dimensional plots of quadratic selection on anthocyanin (ANT), water balance index (WBI), and spectral reflectance at 700 nm (R700) in the Illinois garden.
**Table S1** Number of individuals used in the spectral PLSDA analysis.
**Table S2** Number of individuals included in aster fitness estimation, selection on traits, and QUERCUS analyses (physio, spectra, morpho).
**Table S3** Summary information for traits used in the QUERCUS analysis.
**Table S4** Linear mixed effect (M1a,b) and quadratic mixed effect models (M2a,b) used in selection analysis, with models for garden‐specific and where is the response variable (relative fitness) for the *i* ^th^ observation in the *j* ^th^ block and the kth population and are fixed effect predictors for the traits anthocyanins, relative growth rate, leaf mass per area, water band index, chlorophyll : carotenoid index, leaf thickness, and reflectance at 700 nm, respectively.
**Table S5** Genetic variance (*V*g) and environmental variance (*V*e) in each population for each garden, and broad sense heritability and delta calculated confidence intervals.
Please note: Wiley is not responsible for the content or functionality of any Supporting Information supplied by the authors. Any queries (other than missing material) should be directed to the *New Phytologist* Central Office.

## Data Availability

Data and code are provided on Dryad.com at https://doi.org/10.5061/dryad.xwdbrv1ps.

## References

[nph71003-bib-0001] Adams WW , Demmig‐Adams B . 1995. The xanthophyll cycle and sustained thermal energy dissipation activity in Vinca minor and euonymus Kiautschovicus in winter. Plant, Cell & Environment 18: 117–127.

[nph71003-bib-0002] Ahrens CW , Andrew ME , Mazanec RA , Ruthrof KX , Challis A , Hardy G , Byrne M , Tissue DT , Rymer PD . 2020. Plant functional traits differ in adaptability and are predicted to be differentially affected by climate change. Ecology and Evolution 10: 232–248. 31988725 10.1002/ece3.5890 PMC6972804

[nph71003-bib-0003] Aitken SN , Whitlock MC . 2013. Assisted gene flow to facilitate local adaptation to climate change. Annual Review of Ecology, Evolution, and Systematics 44: 367–388.

[nph71003-bib-0004] Aitken SN , Yeaman S , Holliday JA , Wang T , Curtis‐McLane S . 2008. Adaptation, migration or extirpation: climate change outcomes for tree populations. Evolutionary Applications 1: 95–111. 25567494 10.1111/j.1752-4571.2007.00013.x PMC3352395

[nph71003-bib-0005] Alberto FJ , Aitken SN , Alía R , González‐Martínez SC , Hänninen H , Kremer A , Lefèvre F , Lenormand T , Yeaman S , Whetten R *et al*. 2013. Potential for evolutionary responses to climate change – evidence from tree populations. Global Change Biology 19: 1645–1661. 23505261 10.1111/gcb.12181 PMC3664019

[nph71003-bib-0006] Ashikhmin A , Bolshakov M , Pashkovskiy P , Vereshchagin M , Khudyakova A , Shirshikova G , Kozhevnikova A , Kosobryukhov A , Kreslavski V , Kuznetsov V *et al*. 2023. The adaptive role of carotenoids and Anthocyanins in *Solanum lycopersicum* pigment mutants under high irradiance. Cells 12: 21. 10.3390/cells12212569 PMC10650732 37947647

[nph71003-bib-0007] Austerlitz F , Smouse PE . 2001. Two‐generation analysis of pollen flow across a landscape. III. Impact of adult population structure. Genetical Research 78: 271–280. 11865716 10.1017/s0016672301005341

[nph71003-bib-0008] Ballesta P , Ahmar S , Lobos GA , Mieres‐Castro D , Jiménez‐Aspee F , Mora‐Poblete F . 2022. Heritable variation of foliar spectral reflectance enhances genomic prediction of hydrogen cyanide in a genetically structured population of eucalyptus. Frontiers in Plant Science 13. doi: 10.3389/fpls.2022.871943. PMC9008590 35432412

[nph71003-bib-0116] Barnabás B , Jäger K , Fehér A . 2008. The effect of drought and heat stress on reproductive processes in cereals. Plant, Cell & Environment 31: 11–38. 10.1111/j.1365-3040.2007.01727.x 17971069

[nph71003-bib-0009] Bemmels JB , Anderson JT . 2019. Climate change shifts natural selection and the adaptive potential of the perennial forb *Boechera stricta* in the Rocky Mountains. Evolution 73: 2247–2262. 31584183 10.1111/evo.13854

[nph71003-bib-0010] Browne L , Wright JW , Fitz‐Gibbon S , Gugger PF , Sork VL . 2019. Adaptational lag to temperature in Valley Oak (*Quercus lobata*) can be mitigated by genome‐informed assisted gene flow. Proceedings of the National Academy of Sciences, USA 116: 25179–25185. 10.1073/pnas.1908771116 PMC6911187 31767740

[nph71003-bib-0011] Butler P , Swanston C , Janowiak M , Parker L , St. Pierre M , Brandt L . 2012. Chapter 2: adaptation strategies and approaches. In: Forest adaptation resources: climate change tools and approaches for land managers. En. Tech. Rep. NRS‐87. Newtown Square, PA, USA: U.S. Department of Agriculture, Forest Service, Northern Research Station.

[nph71003-bib-0012] Cavender‐Bares J , Bazzaz FA . 2000. Changes in drought response strategies with ontogeny in *Quercus rubra*: implications for scaling from seedlings to mature trees. Oecologia 124: 8–18. 28308415 10.1007/PL00008865

[nph71003-bib-0013] Cavender‐Bares J . 2019. Diversification, adaptation, and community assembly of the American Oaks (Quercus), a model clade for integrating ecology and evolution. New Phytologist 221: 669–692. 30368821 10.1111/nph.15450

[nph71003-bib-0014] Cavender‐Bares J , Gamon JA , Hobbie SE , Madritch MD , Meireles JE , Schweiger AK , Townsend PA . 2017. Harnessing plant spectra to integrate the biodiversity sciences across biological and spatial scales. American Journal of Botany 104: 966–969. 28724594 10.3732/ajb.1700061

[nph71003-bib-0304] Cavender‐Bares J , Meireles JE , Pinto‐Ledezma J , Reich PB , Schuman MC , Townsend PA , Trowbridge A . 2025. Spectral biology across scales in changing environments. *Preprint*, Life Sciences (February 18). doi: 10.32942/X2H623. PMC12277874 40685754

[nph71003-bib-0015] Chabot BF , Hicks DJ . 1982. The ecology of leaf life spans. Annual Review of Ecology and Systematics 13: 229–259.

[nph71003-bib-0016] Cohen J . 1960. A coefficient of agreement for nominal scales. Educational and Psychological Measurement 20: 37–46.

[nph71003-bib-0017] Copernicus Climate Change Service . 2023. “ERA5 monthly averaged data on single levels from 1940 to present.” Copernicus Climate Change Service (C3S) Climate Data Store (CDS). doi: 10.24381/cds.f17050d7.

[nph71003-bib-0018] Corbin JPM , Best RJ , Garthwaite IJ , Cooper HF , Doughty CE , Gehring CA , Hultine KR , Allan GJ , Whitham TG . 2025. Hyperspectral leaf reflectance detects interactive genetic and environmental effects on tree phenotypes, enabling large‐scale monitoring and restoration planning under climate change. Plant, Cell & Environment 48: 1842–1857. 10.1111/pce.15263 PMC11788971 39497286

[nph71003-bib-0019] Cornelius J . 1994. The effectiveness of plus‐tree selection for yield. Forest Ecology and Management 67: 23–34.

[nph71003-bib-0020] Cornic G . 2000. Drought stress inhibits photosynthesis by decreasing stomatal aperture – not by affecting ATP synthesis. Trends in Plant Science 5: 187–188.

[nph71003-bib-0021] Croft H , Chen JM , Luo X , Bartlett P , Chen B , Staebler RM . 2017. Leaf chlorophyll content as a proxy for leaf photosynthetic capacity. Global Change Biology 23: 3513–3524. 27976452 10.1111/gcb.13599

[nph71003-bib-0022] Curran PJ , Dungan JL , Gholz HL . 1990. Exploring the relationship between reflectance red edge and chlorophyll content in slash pine. Tree Physiology 7: 33–48. 14972904 10.1093/treephys/7.1-2-3-4.33

[nph71003-bib-0023] Demmig‐Adams B , Adams WW III . 2006. Photoprotection in an ecological context: the remarkable complexity of thermal energy dissipation. New Phytologist 172: 11–21. 16945085 10.1111/j.1469-8137.2006.01835.x

[nph71003-bib-0024] Desmond SC , Garner M , Flannery S , Whittemore AT , Hipp AL . 2021. Leaf shape and size variation in bur oaks: an empirical study and simulation of sampling strategies. American Journal of Botany 108: 1540–1554. 34387858 10.1002/ajb2.1705

[nph71003-bib-0025] Dunlap JM , Stettler RF . 2001. Variation in leaf epidermal and stomatal traits of *Populus trichocarpa* from two transects across the Washington cascades. Canadian Journal of Botany 79: 528–536.

[nph71003-bib-0026] Eckert CG , Samis KE , Lougheed SC . 2008. Genetic variation across species' geographical ranges: the central–marginal hypothesis and beyond. Molecular Ecology 17: 1170–1188. 18302683 10.1111/j.1365-294X.2007.03659.x

[nph71003-bib-0027] Etterson JR , Shaw RG . 2001. Constraint to adaptive evolution in response to global warming. Science 294: 151–154. 11588260 10.1126/science.1063656

[nph71003-bib-0028] Etterson JR . 2004. Evolutionary potential of *Chamaecrista fasciculata* in relation to climate change. II. Genetic architecture of three populations reciprocally planted along an environmental gradient in the great plains. Evolution 58: 1459–1471. 15341149 10.1111/j.0014-3820.2004.tb01727.x

[nph71003-bib-0029] Etterson JR , Cornett MW , White MA , Kavajecz LC . 2020. Assisted migration across fixed seed zones detects adaptation lags in two major north American tree species. Ecological Applications 30: e02092. 32058650 10.1002/eap.2092 PMC7534057

[nph71003-bib-0030] Everingham SE , Offord CA , Sabot MEB , Moles AT . 2024. Leaf morphological traits show greater responses to changes in climate than leaf physiological traits and gas exchange variables. Ecology and Evolution 14: e10941. 38510539 10.1002/ece3.10941 PMC10951557

[nph71003-bib-0031] Falconer DS , Mackay TFC . 1996. Introduction to quantitative genetics, 4th edn. Harlow, Essex, UK: Longmans Green.

[nph71003-bib-0032] Fastovich D , Meyers SR , Saupe EE , Williams JW , Dornelas M , Dowding EM , Finnegan S , Huang HM , Jonkers L , Kiessling W *et al*. 2025. Coupled, decoupled, and abrupt responses of vegetation to climate across timescales. Science 389: 64–68. 40608929 10.1126/science.adr6700

[nph71003-bib-0033] Féret J‐B , le Maire G , Jay S , Berveiller D , Bendoula R , Hmimina G , Cheraiet A , Oliveira JC , Ponzoni FJ , Solanki T *et al*. 2019. Estimating leaf mass per area and equivalent water thickness based on leaf optical properties: potential and limitations of physical modeling and machine learning. Remote Sensing of Environment 231(September): 110959.

[nph71003-bib-0034] Féret J‐B , De Boissieu F . 2024. Prospect: an R package to link leaf optical properties with their chemical and structural properties with the leaf model PROSPECT. Journal of Open Source Software 9: 6027.

[nph71003-bib-0105] Fox J , Weisberg S , Price B , Adler D , Bates D , Baud‐Bovy G , Bolker B , Ellison S , Firth D , Friendly M *et al*. 2023. car: companion to applied regression . v.3.1‐2. Released March 30. [WWW document] URL https://cran.r-project.org/web/packages/car/index.html.

[nph71003-bib-0035] Franks SJ , Weber JJ , Aitken SN . 2014. Evolutionary and plastic responses to climate change in terrestrial plant populations. Evolutionary Applications 7: 123–139. 24454552 10.1111/eva.12112 PMC3894902

[nph71003-bib-0036] Galatowitsch S , Frelich L , Phillips‐Mao L . 2009. Regional climate change adaptation strategies for biodiversity conservation in a midcontinental region of North America. Biological Conservation 142: 2012–2022.

[nph71003-bib-0037] Galloway LF , Etterson JR . 2007. Transgenerational plasticity is adaptive in the wild. Science 318: 1134–1136. 18006745 10.1126/science.1148766

[nph71003-bib-0038] Gamache I , Payette S . 2004. Height growth response of tree line black spruce to recent climate warming across the forest‐tundra of Eastern Canada. Journal of Ecology 92: 835–845.

[nph71003-bib-0040] Gamon JA , Huemmrich KF , Wong CYS , Wong CY , Ensminger I , Garrity S , Hollinger DY , Noormets A , Peñuelas J . 2016. A remotely sensed pigment index reveals photosynthetic phenology in evergreen conifers. Proceedings of the National Academy of Sciences, USA 113: 13087–13092. 10.1073/pnas.1606162113 PMC5135292 27803333

[nph71003-bib-0039] Gamon JA , Somers B , Malenovský Z , Middleton EM , Rascher U , Schaepman ME . 2019. Assessing vegetation function with imaging spectroscopy. Surveys in Geophysics 40(3): 489–513. doi: 10.1007/s10712-019-09511-5.

[nph71003-bib-0041] Gauzere J , Teuf B , Davi H , Chevin LM , Caignard T , Leys B , Delzon S , Ronce O , Chuine I . 2020. Where is the optimum? Predicting the variation of selection along climatic gradients and the adaptive value of plasticity. A case study on tree phenology. Evolution Letters 4: 109–123. 32313687 10.1002/evl3.160 PMC7156102

[nph71003-bib-0042] George J‐P , Theroux‐Rancourt G , Rungwattana K , Scheffknecht S , Momirovic N , Neuhauser L , Weißenbacher L , Watzinger A , Hietz P . 2020. Assessing adaptive and plastic responses in growth and functional traits in a 10‐year‐old common garden experiment with Pedunculate Oak (*Quercus robur* L.) suggests that directional selection can drive climatic adaptation. Evolutionary Applications 13: 2422–2438. 33005231 10.1111/eva.13034 PMC7513705

[nph71003-bib-0043] Geyer CJ , Wagenius S , Shaw RG . 2007. Aster models for life history analysis. Biometrika 94: 415–426.

[nph71003-bib-0044] Ghouil H , Sancho‐Knapik D , Ben Mna A , Amimi N , Ammari Y , Escribano R , Alonso‐Forn D , Ferrio JP , Peguero‐Pina JJ , Gil‐Pelegrín E . 2020. Southeastern rear edge populations of *Quercus suber* L. showed two alternative strategies to cope with water stress. Forests 11: 1344.

[nph71003-bib-0045] Givnish T . 1979. On the adaptive significance of leaf form. In: Solbrig OT , Jain S , Johnson GB , Raven PH , eds. Topics in plant population biology. Palgrave, London, UK: Macmillan Education UK. doi: 10.1007/978-1-349-04627-0_17.

[nph71003-bib-0046] Givnish TJ . 1987. Comparative studies of leaf form: assessing the relative roles of selective pressures and phylogenetic constraints. New Phytologist 106: 131–160. doi: 10.1111/j.1469-8137.1987.tb04687.x.

[nph71003-bib-0047] Gould KS . 2004. Nature's Swiss army knife: the diverse protective roles of anthocyanins in leaves. Journal of Biomedicine and Biotechnology 2004: 314–320. 15577195 10.1155/S1110724304406147 PMC1082902

[nph71003-bib-0048] Gould KS , Markham KR , Smith RH , Goris JJ . 2000. Functional role of anthocyanins in the leaves of Quintinia Serrata A. Cunn. Journal of Experimental Botany 51: 1107–1115. 10948238 10.1093/jexbot/51.347.1107

[nph71003-bib-0049] Govindjee . 2004. Chlorophyll a fluorescence: a bit of basics and history. In: Papageorgiou GC , Govindjee , eds. Chlorophyll a fluorescence: a signature of photosynthesis. Springer, Dordrecht, the Netherlands: Springer Netherlands. doi: 10.1007/978-1-4020-3218-9_1.

[nph71003-bib-0050] Grime JP . 2006. Trait convergence and trait divergence in herbaceous plant communities: mechanisms and consequences. Journal of Vegetation Science 17: 255.

[nph71003-bib-0051] Groom PK , Lamont BB , Leighton S , Leighton P , Burrows C . 2004. Heat damage in sclerophylls is influenced by their leaf properties and plant environment. Écoscience 11: 94–101. doi: 10.1080/11956860.2004.11682813.

[nph71003-bib-0106] Gugger PF , Fitz‐Gibbon S , PellEgrini M , Sork VL . 2016. Species‐wide patterns of DNA methylation variation in *Quercus lobata* and their association with climate gradients. Molecular Ecology 25: 1665–1680. 26833902 10.1111/mec.13563

[nph71003-bib-0052] Guo Q . 2012. Incorporating latitudinal and central–marginal trends in assessing genetic variation across species ranges. Molecular Ecology 21: 5396–5403. 23051180 10.1111/mec.12012

[nph71003-bib-0053] Hamrick JL . 2004. Response of forest trees to global environmental changes. Forest Ecology and Management 197: 323–335.

[nph71003-bib-0054] Handler S , Iverson L , Peters E , Scheller RM , Wythers KR , Brandt L , Butler P , Janowiak M , Shannon PD , Swanston C . 2014. Minnesota forest ecosystem vulnerability assessment and synthesis: a report from the northwoods climate change response framework project. In: General technical report. Newtown Square, PA, USA: U.S. Department of Agriculture, Forest Service, Northern Research Station, 1–228. doi: 10.2737/NRS-GTR-133.

[nph71003-bib-0055] Hersbach H , Bell B , Berrisford P . 2023. ERA5 monthly averaged data on single levels from 1940 to present. Copernicus Climate Change Service (C3S) doi: 10.24381/cds.f17050d7.

[nph71003-bib-0056] Hoffmann AA , Sgrò CM , Kristensen TN . 2017. Revisiting Adaptive Potential, Population Size, and Conservation. Trends in Ecology & Evolution 32: 506–517. 28476215 10.1016/j.tree.2017.03.012

[nph71003-bib-0057] Intergovernmental Panel on Climate Change (IPCC) , ed. 2022. Impacts of 1.5°C Global Warming on Natural and Human Systems. In: Global Warming of 1.5°C: IPCC Special Report on Impacts of Global Warming of 1.5°C above Pre‐Industrial Levels in Context of Strengthening Response to Climate Change, Sustainable Development, and Efforts to Eradicate Poverty. Cambridge, UK: Cambridge University Press. doi: 10.1017/9781009157940.005.

[nph71003-bib-0058] Jacquemoud S , Ustin S . 2019. Extraction of leaf traits. In: Leaf optical properties. Cambridge UK: Cambridge University Press. doi: 10.1017/9781108686457.

[nph71003-bib-0059] Kaproth MA , Fredericksen BW , González‐Rodríguez A , Hipp AL , Cavender‐Bares J . 2023. Drought response strategies are coupled with leaf habit in 35 evergreen and deciduous oak (*Quercus*) species across a climatic gradient in the Americas. New Phytologist 239: 888–904. 37282764 10.1111/nph.19019

[nph71003-bib-0060] Kingsolver JG , Hoekstra HE , Hoekstra JM , Berrigan D , Vignieri SN , Hill CE , Hoang A , Gibert P , Beerli P . 2001. The strength of phenotypic selection in natural populations. American Naturalist 157: 245–261. 10.1086/319193 18707288

[nph71003-bib-0061] Knipling EB . 1970. Physical and physiological basis for the reflectance of visible and near‐infrared radiation from vegetation. Remote Sensing of Environment 1: 155–159.

[nph71003-bib-0062] Kothari S , Schweiger AK . 2022. Plant spectra as integrative measures of plant phenotypes. Journal of Ecology 110: 2536–2554.

[nph71003-bib-0063] Kozlowski TT , Pallardy SG . 2002. Acclimation and adaptive responses of woody plants to environmental stresses. Botanical Review 68: 270–334.

[nph71003-bib-0064] Kuhn M , Wing J , Weston S . 2023. Caret: classification and regression training. v.6.0‐94. [WWW document] URL https://cran.r‐project.org/web/packages/caret/.

[nph71003-bib-0065] Lande R , Arnold SJ . 1983. The measurement of selection on correlated characters. Evolution 37: 1210. 28556011 10.1111/j.1558-5646.1983.tb00236.x

[nph71003-bib-0066] Lawlor DAVIDW . 2002. Limitation to photosynthesis in water‐stressed leaves: stomata vs. metabolism and the role of ATP. Annals of Botany 89: 871–885. 12102513 10.1093/aob/mcf110 PMC4233810

[nph71003-bib-0067] Lee J‐Y , Marotzke J , Bala G , Cao L , Corti S , Dunne JP , Engelbrecht F , Fischer E , Fyfe JC , Jones C *et al*. 2021. Chapter 4: future global climate: scenario‐based projections and near‐term information. In: Climate change 2021: the physical science basis. Contribution of working group I to the sixth assessment report of the intergovernmental panel on climate change. Cambridge, UK: Cambridge University Press. doi: 10.1017/9781009157896.

[nph71003-bib-0117] Li S , Fleisher DH , Barnaby JY . 2024. Quantifying the impact of climate change and extreme heat on rice in the United States. Agricultural and Forest Meteorology 355: 110145.

[nph71003-bib-0068] Lush JL . 1937. Animal breeding plans. Ames, IA, USA: Iowa State College Press.

[nph71003-bib-0070] McDowell N , Pockman WT , Allen CD , Breshears DD , Cobb N , Kolb T , Plaut J , Sperry J , West A , Williams DG *et al*. 2008. Mechanisms of plant survival and mortality during drought: why do some plants survive while others succumb to drought? New Phytologist 178: 719–739. 18422905 10.1111/j.1469-8137.2008.02436.x

[nph71003-bib-0069] McDowell NG , Sapes G , Pivovaroff A , Adams HD , Allen CD , Anderegg WRL , Arend M , Breshears DD , Brodribb T , Choat B *et al*. 2022. Mechanisms of woody‐plant mortality under rising drought, CO_2_ and vapour pressure deficit. Nature Reviews Earth & Environment 3: 294–308. doi: 10.1038/s43017-022-00272-1.

[nph71003-bib-0071] Meireles JE , Beulke A , Borkowski DS , Romero‐Severson J , Cavender‐Bares J . 2017. Balancing selection maintains diversity in a cold tolerance gene in broadly distributed live oaks. Genome 60: 762–769. 28683204 10.1139/gen-2016-0208

[nph71003-bib-0072] Meireles JE , Schweiger AK , Cavender‐Bares J . 2021. Package ‘Spectrolab’.

[nph71003-bib-0073] Mitchell‐Olds T , Rutledge JJ . 1986. Quantitative genetics in natural plant populations: a review of the theory. The American Naturalist 127: 379–402.

[nph71003-bib-0074] Mobasheri MR , Fatemi SB . 2013. Leaf equivalent water thickness assessment using reflectance at optimum wavelengths. Theoretical and Experimental Plant Physiology 25: 196–202.

[nph71003-bib-0075] Mousseau TA , Fox CW . 1998. Maternal effects as adaptations. Oxford, UK: Oxford University Press.

[nph71003-bib-0076] Ostrowsky L , Rea L , Hernández‐Leal M , Mohn R , Garner M , Worcester L , Lapadat C , Clevenger J , Myers Z , Sanmartin KP *et al*. 2026. Evidence for adaptive variation in *Quercus macrocarpa* (L.) leaf morphology from a reciprocal transplant experiment across a latitudinal gradient. Forest Ecology and Management 600(January): 123292.

[nph71003-bib-0077] Penuelas J , Pinol J , Ogaya R , Filella I . 1997. Estimation of plant water concentration by the reflectance water index WI (R900/R970). International Journal of Remote Sensing 18: 2869–2875.

[nph71003-bib-0078] Pu R , Foschi L , Gong P . 2004. Spectral feature analysis for assessment of water status and health level in coast live oak (*Quercus agrifolia*) leaves. International Journal of Remote Sensing 25: 4267–4286.

[nph71003-bib-0079] R Core Team . 2023. R: a language and environment for statistical computing. Vienna, Austria: R Foundation for Statistical Computing.

[nph71003-bib-0080] Ramírez‐Valiente JA , Etterson JR , Deacon NJ , Cavender‐Bares J . 2019. Evolutionary potential varies across populations and traits in the neotropical oak *Quercus oleoides* . Tree Physiology 39: 427–439. 30321394 10.1093/treephys/tpy108

[nph71003-bib-0081] Ramírez‐Valiente JA , Koehler K , Cavender‐Bares J . 2015. Climatic origins predict variation in photoprotective leaf pigments in response to drought and low temperatures in live oaks (Quercus Series Virentes). Tree Physiology 35: 521–534. 25939867 10.1093/treephys/tpv032

[nph71003-bib-0082] Rea LMS , Ostrowsky L , Mohn R , Garner M , Worcester L , Lapadat C , McCarthy HR , Hipp AL , Cavender Bares J . 2024. Genetically based variation in fitness and carbon assimilation among bur oak populations. *bioRxiv*, 2024.10.30.620350. doi: 10.1101/2024.10.30.620350.

[nph71003-bib-0083] Ribicoff G , Garner M , Pham K , Althaus KN , Cavender‐Bares J , Crowl AA , Gray S , Gugger P , Hahn M , Liao S *et al*. 2025. Introgression, phylogeography, and genomic species cohesion in the eastern north American white oak Syngameon. Molecular Ecology n/a: e17822. 10.1111/mec.17822 40491223

[nph71003-bib-0084] Sapes G , Lapadat C , Schweiger AK , Juzwik J , Montgomery R , Gholizadeh H , Townsend PA , Gamon JA , Cavender‐Bares J . 2022. Canopy spectral reflectance detects oak wilt at the landscape scale using phylogenetic discrimination. Remote Sensing of Environment 273(May): 112961.

[nph71003-bib-0085] Schlaepfer DR , Braschler B , Rusterholz H‐P , Baur B . 2018. Genetic effects of anthropogenic habitat fragmentation on remnant animal and plant populations: a meta‐analysis. Ecosphere 9: e02488.

[nph71003-bib-0086] Schuepp PH . 1993. Tansley review no. 59 leaf boundary layers. New Phytologist 125: 477–507. 33874584 10.1111/j.1469-8137.1993.tb03898.x

[nph71003-bib-0087] Schweiger AK , Cavender‐Bares J , Townsend PA , Hobbie SE , Madritch MD , Wang R , Tilman D , Gamon JA . 2018. Plant spectral diversity integrates functional and phylogenetic components of biodiversity and predicts ecosystem function. Nature Ecology & Evolution 2: 976–982. 29760440 10.1038/s41559-018-0551-1

[nph71003-bib-0088] Serbin SP , Townsend PA . 2020. Scaling functional traits from leaves to canopies. In: Cavender‐Bares J , Gamon JA , Townsend PA , eds. Remote sensing of plant biodiversity. Springer, Cham, Switzerland: Springer International Publishing. doi: 10.1007/978-3-030-33157-3_3.

[nph71003-bib-0089] Shaw G , Platenkamp GAJ , Shaw FH , Podolsky RH . 1994. Quantitative genetics of response to competitors in *Nemophila menziesii*: a field experiment. Genetics 139: 397–406. 10.1093/genetics/139.1.397 PMC1206336 7705640

[nph71003-bib-0090] Shaw RG , Shaw FH . 1994. Quercus quantitative genetics software. [WWW document] URL https://cbs.umn.edu/eeb/about‐eeb/helpful‐links/quercus‐quantitative‐genetics‐software.

[nph71003-bib-0091] Shaw RG . 1987. Maximum‐likelihood approaches applied to quantitative genetics of natural populations. Evolution 41: 812–826. 28564352 10.1111/j.1558-5646.1987.tb05855.x

[nph71003-bib-0092] Shaw RG , Etterson JR . 2012. Rapid climate change and the rate of adaptation: insight from experimental quantitative genetics. New Phytologist 195: 752–765. 22816320 10.1111/j.1469-8137.2012.04230.x

[nph71003-bib-0093] Shaw RG , Geyer CJ , Wagenius S , Hangelbroek HH , Etterson JR . 2008. Unifying life‐history analyses for inference of fitness and population growth. American Naturalist 172: E35–E47. 10.1086/588063 18500940

[nph71003-bib-0094] Sheffield J , Wood EF . 2008. Projected changes in drought occurrence under future global warming from multi‐model, multi‐scenario, IPCC Ar4 simulations. Climate Dynamics 31: 79–105.

[nph71003-bib-0095] Sims DA , Gamon JA . 2002. Relationships between leaf pigment content and spectral reflectance across a wide range of species, leaf structures and developmental stages. Remote Sensing of Environment 81: 337–354.

[nph71003-bib-0096] Sims DA , Gamon JA . 2003. Estimation of vegetation water content and photosynthetic tissue area from spectral reflectance: a comparison of indices based on liquid water and chlorophyll absorption features. Remote Sensing of Environment 84: 526–537.

[nph71003-bib-0097] Singhal S , Wrath J , Rabosky DL . 2022. Genetic variability and the ecology of geographic range: a test of the central‐marginal hypothesis in Australian Scincid lizards. Molecular Ecology 31: 4242–4253. 35779002 10.1111/mec.16589 PMC9545263

[nph71003-bib-0098] Sork VL , Davis FW , Smouse PE , Apsit VJ , Dyer RJ , Fernandez‐M JF , Kuhn B . 2002. Pollen movement in declining populations of California Valley oak, *Quercus lobata*: where have all the fathers gone? Molecular Ecology 11: 1657–1668. 12207717 10.1046/j.1365-294x.2002.01574.x

[nph71003-bib-0099] Spafford L , le Maire G , MacDougall A , de Boissieu F , Féret J‐B . 2021. Spectral subdomains and prior estimation of leaf structure improves PROSPECT inversion on reflectance or transmittance alone. Remote Sensing of Environment 252(January): 112176.

[nph71003-bib-0100] Springer KR , Wang R , Gamon JA . 2017. Parallel seasonal patterns of photosynthesis, fluorescence, and reflectance indices in boreal trees. Remote Sensing 9: 691.

[nph71003-bib-0107] Stefanski A , Butler EE , Williams LJ , Bermudez R , Guzmán QJA , Larson A , Townsend PA , Montgomery R , Cavender‐Bares J , Reich PB . 2025. All the light we cannot see: climate manipulations leave short and long‐term imprints in spectral reflectance of trees. Ecology 106 (May): e70048. 40369965 10.1002/ecy.70048 PMC12079083

[nph71003-bib-0101] Thornthwaite CW . 1948. An approach toward a rational classification of climate. Geographical Review 38: 55–94.

[nph71003-bib-0102] Travis J . 1989. The role of optimizing selection in natural populations. Annual Review of Ecology and Systematics 20: 279–296.

[nph71003-bib-0103] USA National Phenology Network . 2025. Daily temperature accumulations – 32 base temp. University of Arizona Research Data Repository. doi: 10.5066/F7SN0723.

[nph71003-bib-0104] Vandewoestijne S , Schtickzelle N , Baguette M . 2008. Positive correlation between genetic diversity and fitness in a large, well‐connected metapopulation. BMC Biology 6: 46. 18986515 10.1186/1741-7007-6-46 PMC2587462

[nph71003-bib-0205] Verhoeven A . 2014. Sustained energy dissipation in winter evergreens. New Phytologist 201: 57–65.

[nph71003-bib-0118] Vollenweider P , Günthardt‐Goerg MS . 2005. Diagnosis of abiotic and biotic stress factors using the visible symptoms in foliage. Environmental Pollution, Forests Under Changing Climate, Enhanced UV and Air Pollution 137: 455–465. 10.1016/j.envpol.2005.01.032 16005758

[nph71003-bib-0206] Wang Z , Huang H , Wang H , Peñuelas J , Sardans J , Niinemets Ü , Niklas KJ , Li Y , Xie J , Wright IJ . 2022. Leaf water content contributes to global leaf trait relationships. Nature Communications 13: 5525. 10.1038/s41467-022-32784-1 PMC9492732 36130948

[nph71003-bib-0207] Warwell MV , Shaw RG . 2017. Climate‐related genetic variation in a threatened tree species, *Pinus albicaulis* . American Journal of Botany 104: 1205–1218. 29756223 10.3732/ajb.1700139

[nph71003-bib-0108] Willi Y , Van Buskirk J , Hoffmann AA . 2006. Limits to the adaptive potential of small populations. Annual Review of Ecology, Evolution, and Systematics 37: 433–458.

[nph71003-bib-0109] Wong CYS , D'Odorico P , Arain MA , Ensminger I . 2020. Tracking the phenology of photosynthesis using carotenoid‐sensitive and near‐infrared reflectance vegetation indices in a temperate evergreen and mixed deciduous forest. New Phytologist 226: 1682–1695. 32039477 10.1111/nph.16479

[nph71003-bib-0110] Wong CYS , D'Odorico P , Bhathena Y , Arain MA , Ensminger I . 2019. Carotenoid based vegetation indices for accurate monitoring of the phenology of photosynthesis at the leaf‐scale in deciduous and evergreen trees. Remote Sensing of Environment 233: 111407.

[nph71003-bib-0111] Wright IJ , Dong N , Maire V , Prentice IC , Westoby M , Díaz S , Gallagher RV , Jacobs BF , Kooyman R , Law EA *et al*. 2017. Global climatic drivers of leaf size. Science 357: 917–921. 28860384 10.1126/science.aal4760

[nph71003-bib-0112] Wright IJ , Reich PB , Westoby M , Ackerly DD , Baruch Z , Bongers F , Cavender‐Bares J , Chapin T , Cornelissen JH , Diemer M *et al*. 2004. The worldwide leaf economics spectrum. Nature 428: 821–827. 15103368 10.1038/nature02403

[nph71003-bib-0113] Wright IJ , Westoby M , Reich PB . 2002. Convergence towards higher leaf mass per area in dry and nutrient‐poor habitats has different consequences for leaf life span. Journal of Ecology 90: 534–543.

[nph71003-bib-0114] Wujeska A , Bossinger G , Tausz M . 2013. Responses of foliar antioxidative and photoprotective defence systems of trees to drought: a meta‐analysis. Tree Physiology 33: 1018–1029. 24178981 10.1093/treephys/tpt083

[nph71003-bib-0208] Yakovlev I , Fossdal CG , Skrøppa T , Olsen JE , Jahren AH , Johnsen Ø . 2012. An adaptive epigenetic memory in conifers with important implications for seed production. Seed Science Research 22: 63–76.

[nph71003-bib-0115] Young SS , Young JS . 2025. Decreasing snow cover and increasing temperatures are accelerating in New England, USA, with long‐term implications. Climate 13: 246.

