# Supplementary material for: Greater climate change adaptation potential in populations of Quercus macrocarpa at edges of latitudinal gradient
Source: New Phytol. 2026 Feb 16;250(2):1313–29. doi: 10.1111/nph.71003 (PMC13001018; doi:10.1111/nph.71003)
Supplement: Supplementary file 1 — Fig. S1 Map of collections for each population, overlayed over species distribution for Quercus macrocarpa from United States Geological Survey. Fig. S2 Temperature and precipitation in Vallonia, Indiana Department of Natural Resources Nursery, where trees were germinated and grew from 2018 to 2021. Fig. S3 Box plot of heights for each population in each garden in 2023 and Percent mortality in 2023 in each population in each garden. Fig. S4 Correlation matrix of traits used in selection analysis. Fig. S5 Two dimensional plots of quadratic selection on anthocyanin (ANT), water balance index (WBI), and spectral reflectance at 700 nm (R700) in the Illinois garden. Table S1 Number of individuals used in the spectral PLSDA analysis. Table S2 Number of individuals included in aster fitness estimation, selection on traits, and QUERCUS analyses (physio, spectra, morpho). Table S3 Summary information for traits used in the QUERCUS analysis. Table S4 Linear mixed effect (M1a,b) and quadratic mixed effect models (M2a,b) used in selection analysis, with models for garden‐specific and where is the response variable (relative fitness) for the i th observation in the j th block and the kth population and are fixed effect predictors for the traits anthocyanins, relative growth rate, leaf mass per area, water band index, chlorophyll : carotenoid index, leaf thickness, and reflectance at 700 nm, respectively. Table S5 Genetic variance (Vg) and environmental variance (Ve) in each population for each garden, and broad sense heritability and delta calculated confidence intervals. Please note: Wiley is not responsible for the content or functionality of any Supporting Information supplied by the authors. Any queries (other than missing material) should be directed to the New Phytologist Central Office. [file NPH-250-1313-s001.pdf]

Supporting information

New Phytologist Supporting Information

Article title: Greater climate change adaptation potential in populations of Quercus macrocarpa at edges of latitudinal gradient

Author names: Rea, Lucy M.S.[1], Ostrowsky, Laura[1,2], Mohn, Rebekah A.[3,4], Garner, Mira[3,5], Worcester, Lindsey[3], Lapadat, Cathleen[1], McCarthy, Heather R.[6], Hipp, Andrew L.[3], Cavender Bares, Jeannine[1,2]

Article acceptance date: 20 January 2026

The following Supporting Information is available for this article:

Tables

Table S1. Number of individuals used in the spectral PLSDA analysis.

Garden	Population	Count
OK	OK	61
OK	IL	48
OK	MN	42
IL	OK	179
IL	IL	183
IL	MN	180
MN	OK	157
MN	IL	161
MN	MN	165

Table S2. Number of individuals included in aster fitness estimation, selection on traits, and QUERCUS analyses (physio, spectra, morpho).

**population**	**garden**	**mother_id**	**aster count**	**trait count**	**physio count**	**spectra count**	**morpho count**

IL	IL	IL_BFP_11	10	9	9	9	9
IL	IL	IL_BFP_12	9	8	9	9	9
IL	IL	IL_BFP_13	10	10	10	10	10
IL	IL	IL_DAW_36	10	10	10	10	10
IL	IL	IL_DFP_10	10	9	9	9	9
IL	IL	IL_DFP_6	10	7	7	7	7
IL	IL	IL_GFP_19	10	8	8	8	8
IL	IL	IL_JAM_33	10	9	9	9	9
IL	IL	IL_MOR_1	10	4	4	4	4
IL	IL	IL_MOR_26	10	10	10	10	10
IL	IL	IL_MOR_27	10	10	10	10	10
IL	IL	IL_MOR_28	10	9	9	9	9
IL	IL	IL_MOR_30	10	10	10	10	10
IL	IL	IL_MOR_31	10	10	10	10	10
IL	IL	IL_QBP_37	10	10	10	10	10
IL	IL	IL_QBP_38	10	9	9	9	9
IL	IL	IL_QBP_39	10	10	10	10	10
IL	IL	IL_QBP_40	10	10	10	10	10
IL	IL	IL_QBP_41	10	10	10	10	10
IL	IL	IL_QBP_42	10	10	10	10	10
IL	MN	IL_BFP_11	10	8	8	8	8
IL	MN	IL_BFP_12	10	7	7	7	7
IL	MN	IL_BFP_13	10	6	6	6	6
IL	MN	IL_DAW_36	10	8	8	8	8
IL	MN	IL_DFP_10	10	7	7	7	7
IL	MN	IL_DFP_6	10	9	9	9	9
IL	MN	IL_GFP_19	10	7	7	7	7
IL	MN	IL_JAM_33	10	10	10	10	10
IL	MN	IL_MOR_1	8	6	6	6	6
IL	MN	IL_MOR_26	10	10	10	10	10
IL	MN	IL_MOR_27	12	8	8	8	8
IL	MN	IL_MOR_28	11	9	8	8	9
IL	MN	IL_MOR_30	10	9	9	9	9
IL	MN	IL_MOR_31	10	7	7	7	7
IL	MN	IL_QBP_37	10	9	9	9	9
IL	MN	IL_QBP_38	10	8	8	8	8
IL	MN	IL_QBP_39	10	7	8	8	8
IL	MN	IL_QBP_40	10	9	9	9	9
IL	MN	IL_QBP_41	10	10	10	10	10
IL	MN	IL_QBP_42	10	7	7	7	7
IL	OK	IL_BFP_11	10	3	3	3	3
IL	OK	IL_BFP_12	10	4	4	4	4
IL	OK	IL_BFP_13	10	4	4	4	4
IL	OK	IL_DAW_36	10	NA	NA	NA	NA
IL	OK	IL_DFP_10	10	2	2	2	2

IL	OK	IL_DFP_6	10	3	3	3	3
IL	OK	IL_GFP_19	10	4	4	4	4
IL	OK	IL_JAM_33	10	3	3	3	3
IL	OK	IL_MOR_1	10	2	2	2	2
IL	OK	IL_MOR_26	10	3	3	3	3
IL	OK	IL_MOR_27	10	2	2	2	2
IL	OK	IL_MOR_28	10	2	2	2	2
IL	OK	IL_MOR_30	10	3	3	3	3
IL	OK	IL_MOR_31	10	1	1	1	1
IL	OK	IL_QBP_37	10	1	2	2	2
IL	OK	IL_QBP_38	10	2	2	2	2
IL	OK	IL_QBP_39	10	2	2	2	2
IL	OK	IL_QBP_40	10	1	1	1	1
IL	OK	IL_QBP_41	10	3	3	3	3
IL	OK	IL_QBP_42	10	2	2	2	2
MN	IL	MN_BUN_52	10	10	10	10	10
MN	IL	MN_BUN_53	10	8	8	8	8
MN	IL	MN_CAR_8	10	7	7	7	7
MN	IL	MN_CAR_9	10	8	8	8	8
MN	IL	MN_CDR_1	10	10	10	10	10
MN	IL	MN_CDR_28	10	9	9	9	9
MN	IL	MN_CDR_35	10	10	10	10	10
MN	IL	MN_CDR_38	10	8	8	8	8
MN	IL	MN_CDR_39	10	10	10	10	10
MN	IL	MN_COO_16	10	10	10	10	10
MN	IL	MN_COO_17	10	10	10	10	10
MN	IL	MN_COO_18	10	8	8	8	8
MN	IL	MN_CRO_13	10	9	9	9	9
MN	IL	MN_CRO_14	10	8	8	8	8
MN	IL	MN_CRO_15	10	7	7	7	7
MN	IL	MN_GEO_49	10	10	10	10	10
MN	IL	MN_GEO_50	10	10	10	10	10
MN	IL	MN_GEO_51	10	9	9	9	9
MN	IL	MN_LOC_55	10	9	9	9	9
MN	IL	MN_LOC_56	10	10	10	10	10
MN	MN	MN_BUN_52	10	8	8	8	8
MN	MN	MN_BUN_53	10	9	9	9	9
MN	MN	MN_CAR_8	10	8	8	8	8
MN	MN	MN_CAR_9	9	8	8	8	8

MN	MN	MN_CDR_1	10	8	8	8	8
MN	MN	MN_CDR_28	10	7	7	7	7
MN	MN	MN_CDR_35	10	7	7	7	7
MN	MN	MN_CDR_38	10	9	9	9	9
MN	MN	MN_CDR_39	10	10	10	10	10
MN	MN	MN_COO_16	11	8	8	8	8
MN	MN	MN_COO_17	10	6	6	6	6
MN	MN	MN_COO_18	9	7	7	7	7
MN	MN	MN_CRO_13	10	8	8	8	8
MN	MN	MN_CRO_14	10	8	8	8	8
MN	MN	MN_CRO_15	10	9	9	9	9
MN	MN	MN_GEO_49	10	9	9	9	9
MN	MN	MN_GEO_50	10	10	10	10	10
MN	MN	MN_GEO_51	10	10	10	10	10
MN	MN	MN_LOC_55	10	8	8	8	8
MN	MN	MN_LOC_56	10	7	8	8	8
MN	OK	MN_BUN_52	10	3	3	3	3
MN	OK	MN_BUN_53	10	3	3	3	3
MN	OK	MN_CAR_8	10	NA	NA	NA	NA
MN	OK	MN_CDR_1	10	2	2	2	2
MN	OK	MN_CDR_28	10	NA	NA	NA	NA
MN	OK	MN_CDR_35	10	2	2	2	2
MN	OK	MN_CDR_38	10	3	4	4	4
MN	OK	MN_CDR_39	10	1	1	1	1
MN	OK	MN_COO_16	10	3	3	3	3
MN	OK	MN_COO_17	10	5	5	5	5
MN	OK	MN_COO_18	10	2	2	2	2
MN	OK	MN_CRO_13	10	1	2	2	2
MN	OK	MN_CRO_14	10	1	2	2	2
MN	OK	MN_CRO_15	10	3	3	3	3
MN	OK	MN_GEO_49	10	2	2	2	2
MN	OK	MN_GEO_50	10	1	1	1	1
MN	OK	MN_GEO_51	10	2	3	3	3
MN	OK	MN_LOC_55	10	2	2	2	2
MN	OK	MN_LOC_56	10	1	1	1	1
OK	IL	OK_DRA_21	10	10	10	10	10
OK	IL	OK_DRA_22	10	7	7	7	7
OK	IL	OK_DRA_23	10	9	9	9	9

OK	IL	OK_DRA_24	10	7	7	7	7
OK	IL	OK_DRA_25	10	10	10	10	10
OK	IL	OK_DRA_26	10	10	10	10	10
OK	IL	OK_DRA_27	10	10	10	10	10
OK	IL	OK_EDW_10	3	3	3	3	3
OK	IL	OK_EDW_12	12	11	11	11	11
OK	IL	OK_EDW_13	10	10	10	10	10
OK	IL	OK_EDW_14	10	10	10	10	10
OK	IL	OK_EDW_15	10	10	10	10	10
OK	IL	OK_EDW_16	11	8	8	8	8
OK	IL	OK_JIM_29	3	3	3	3	3
OK	IL	OK_KES_28	5	4	4	4	4
OK	IL	OK_MON_17	5	5	5	5	5
OK	IL	OK_MON_18	10	8	8	8	8
OK	IL	OK_MON_19	3	3	3	3	3
OK	IL	OK_MON_6	10	7	7	7	7
OK	IL	OK_OLI_20	10	10	10	10	10
OK	IL	OK_WOO_2	5	5	5	5	5
OK	IL	OK_WOO_4	3	2	2	2	2
OK	IL	OK_WOO_7	10	6	7	7	7
OK	IL	OK_WOO_9	10	10	10	10	10
OK	MN	OK_DRA_21	10	9	9	9	9
OK	MN	OK_DRA_22	10	8	8	8	8
OK	MN	OK_DRA_23	11	9	9	9	9
OK	MN	OK_DRA_24	10	10	10	10	10
OK	MN	OK_DRA_25	10	7	7	7	7
OK	MN	OK_DRA_26	10	8	8	8	8
OK	MN	OK_DRA_27	11	8	8	8	8
OK	MN	OK_EDW_10	3	3	3	3	3
OK	MN	OK_EDW_12	12	8	8	8	8
OK	MN	OK_EDW_13	10	6	7	7	7
OK	MN	OK_EDW_14	10	10	10	10	10
OK	MN	OK_EDW_15	10	8	8	8	8

OK	MN	OK_EDW_16	11	4	4	4	4
OK	MN	OK_JIM_29	3	3	3	3	3
OK	MN	OK_KES_28	4	4	4	4	4
OK	MN	OK_MON_17	5	5	5	5	5
OK	MN	OK_MON_18	9	7	7	7	7
OK	MN	OK_MON_19	3	3	3	3	3
OK	MN	OK_MON_6	10	2	2	2	2
OK	MN	OK_OLI_20	10	9	9	9	9
OK	MN	OK_WOO_2	5	4	4	4	4
OK	MN	OK_WOO_4	3	3	3	3	3
OK	MN	OK_WOO_7	10	9	9	9	9
OK	MN	OK_WOO_9	10	8	9	9	9
OK	OK	OK_DRA_21	10	4	4	4	4
OK	OK	OK_DRA_22	10	2	2	2	2
OK	OK	OK_DRA_23	10	4	4	4	4
OK	OK	OK_DRA_24	10	2	2	2	2
OK	OK	OK_DRA_25	10	1	1	1	1
OK	OK	OK_DRA_26	10	4	4	4	4
OK	OK	OK_DRA_27	10	4	4	4	4
OK	OK	OK_EDW_10	3	NA	NA	NA	NA
OK	OK	OK_EDW_12	12	1	1	1	1
OK	OK	OK_EDW_13	10	3	3	3	3
OK	OK	OK_EDW_14	10	5	5	5	5
OK	OK	OK_EDW_15	10	2	2	2	2
OK	OK	OK_EDW_16	11	5	5	5	5
OK	OK	OK_JIM_29	3	NA	NA	NA	NA
OK	OK	OK_KES_28	5	1	1	1	1
OK	OK	OK_MON_17	5	4	4	4	4
OK	OK	OK_MON_18	10	4	4	4	4
OK	OK	OK_MON_19	3	2	2	2	2
OK	OK	OK_MON_6	10	2	2	2	2
OK	OK	OK_OLI_20	10	2	2	2	2
OK	OK	OK_WOO_2	5	2	2	2	2
OK	OK	OK_WOO_4	3	NA	NA	NA	NA
OK	OK	OK_WOO_7	10	4	4	4	4

OK	OK	OK_WOO_9	10	3	3	3	3

Table S3. Summary information for traits used in the QUERCUS analysis.

**Trait**	**Abbreviation**	**Equation**	**Biological interpretation**	**Data source**
Relative growth rate	RGR		Higher values indicate greater change in stem volume over time	measured
Leaf mass per area	LMA		Higher values indicate thicker or denser leaves per unit area of the leaves	spectral
Leaf thickness			Higher values indicate thicker leaves	measured
Anthocyanin	ANT		Higher values indicate greater levels of anthocyanins in leaves	spectral
Water band index	WBI	$\frac{R970}{R900}$	Higher values indicate greater leaf water concentration	spectral
Chlorophyll:carotenoid index	CCI	$\frac{R531-R645}{R531+R645}$	Higher values indicate higher chlorophyll levels	spectral
Reflectance at 700 nm	R700		Higher values indicate higher photosynthesis	spectral

Reflectance at 900 nm	R900		Higher values indicate healthy leaves with little to no damage to cell walls	spectral
Reflectance at 1400 nm	R1400		Higher values indicate lower water in leaf	spectral

Table S4

Linear mixed effect (M1a,b) and quadratic mixed effect models (M2a,b) used in selection analysis, with models for garden-specific and where is the response variable (relative fitness) for the ith observation in the jth block and the kth population. and are fixed effect predictors for the traits anthocyanins, relative growth rate, leaf mass per area, water band index, chlorophyll:carotenoid index, leaf thickness, and reflectance at 700 nm, respectively. … are fixed effect coefficients for the predictors. is the random effect for block j. is the random effect for population k. is the residual error. For models M1b and M2b, the formula is identical except they do not have the k term for the population effect, since the populations are being analyzed individually.

Model ID	Model equation	Output from model	Analysis
M1a	$Y_{ijk} = \beta 1 \times A_{ijk} + \beta 2 \times R_{ijk} + \beta 3 \times L_{ijk} + \beta 4 \times W_{ijk} + \beta 5 \times C_{ijk} + \beta 6 \times T_{ijk} + \beta 7 \times X_{ijk} + b_j + p_k + \varepsilon_{ijk}$	β, S	Garden-specific
M2a	$Y_{ijk} = \beta 1 \times A^2{}_{ijk} + \beta 2 \times R^2{}_{ijk} + \beta 3 \times L^2{}_{ijk} + \beta 4 \times W^2{}_{ijk} + \beta 5 \times C^2{}_{ijk} + \beta 6 \times T^2{}_{ijk} + \beta 7 \times X^2{}_{ijk} + b_j + p_k + \varepsilon_{ijk}$	γ	Garden-specific
M1b	$Y_{ij} = \beta 1 \times A_{ij} + \beta 2 \times R_{ij} + \beta 3 \times L_{ij} + \beta 4 \times W_{ij} + \beta 5 \times C_{ij} + \beta 6 \times T_{ij} + \beta 7 \times X_{ij} + b_j + \varepsilon_{ij}$	β, S	Population-specific
M2b	$Y_{ij} = \beta 1 \times A^2{}_{ij} + \beta 2 \times R^2{}_{ij} + \beta 3 \times L^2{}_{ij} + \beta 4 \times W^2{}_{ij} + \beta 5 \times C^2{}_{ij} + \beta 6 \times T^2{}_{ij} + \beta 7 \times X^2{}_{ij} + b_j + \varepsilon_{ij}$	γ	Population-specific

Table S5. Genetic variance (V_g) and environmental variance (V_e) in each population for each garden, and broad sense heritability and delta calculated confidence intervals.. Trait were grouped for QUERCUS analysis into *morphological traits (morpho)* (leaf mass per area (LMA), relative growth rate (RGR), and leaf thickness), *physiological traits (physio)* (anthocyanins (ANT), chlorophyll:carotenoid index (CCI), and water band index (WBI)), and *spectral reflectance bands (spectra)* at 700, 900, and 1400 nm. Significance at the $p<0.05$ level calculated with likelihood ratio tests is indicated by boldface.

Population	Garden	Trait	Analysis grouping	V_g	V_g std. error	V_e	V_e std. error	H^2	Lower CI	Upper CI
**OK**	**OK**	**LMA**	**morpho**	**2.57547**	**0.718**	**0.06211**	**0.7032**	**0.557**	**0.219**	**0.895**
**OK**	**IL**	**LMA**	**morpho**	**3.5483**	**0.7837**	**5.40747**	**0.6794**	**0.312**	**-0.660**	**1.284**
**OK**	**MN**	**LMA**	**morpho**	**13.9842**	**0.194**	**6.20837**	**0.182**	**0.580**	**0.580**	**0.580**
**IL**	**OK**	**LMA**	**morpho**	**1.57604**	**0.6227**	**2.15289**	**0.6487**	**0.725**	**0.725**	**0.725**
**IL**	**IL**	**LMA**	**morpho**	**8.19751**	**4.5249**	**6.52254**	**0.5483**	**0.354**	**0.119**	**0.589**
IL	MN	LMA	morpho	0.16226	0.12112	2.04166	0.13243	0.296	0.296	0.296
**MN**	**OK**	**LMA**	**morpho**	**1.14645**	**0**	**1.70781**	**0.3353**	**0.355**	**0.355**	**0.355**
**MN**	**IL**	**LMA**	**morpho**	**3.81885**	**1.0433**	**7.31028**	**0.8681**	**0.051**	**0.051**	**0.051**
**MN**	**MN**	**LMA**	**morpho**	**0.93762**	**0.2004**	**1.56773**	**0.1793**	**0.395**	**0.395**	**0.395**
**OK**	**OK**	**RGR**	**morpho**	**0.36863**	**1.8426**	**0.92966**	**1.591**	**0.343**	**0.261**	**0.425**
**OK**	**IL**	**RGR**	**morpho**	**1.36904**	**2.4067**	**1.17298**	**2.1876**	**0.667**	**-0.937**	**2.272**
**OK**	**MN**	**RGR**	**morpho**	**0.23571**	**7.4139**	**0.45875**	**6.2613**	**0.443**	**0.443**	**0.443**
**IL**	**OK**	**RGR**	**morpho**	**0.02449**	**2.652**	**0.9385**	**2.5464**	**0.052**	**0.052**	**0.052**
**IL**	**IL**	**RGR**	**morpho**	**0.76565**	**0.5853**	**1.68964**	**3.8601**	**0.244**	**0.097**	**0.391**
IL	MN	RGR	morpho	0.05547	0.42205	0.56122	0.46764	0.207	0.207	0.207
**MN**	**OK**	**RGR**	**morpho**	**0**	**1.0652**	**1.39591**	**1.1043**	**0.000**	**0.000**	**0.000**
**MN**	**IL**	**RGR**	**morpho**	**2.04932**	**2.7666**	**1.02131**	**2.5564**	**0.217**	**0.217**	**0.217**
**MN**	**MN**	**RGR**	**morpho**	**0.30165**	**0.6793**	**0.39168**	**0.6224**	**0.228**	**0.228**	**0.228**
**OK**	**OK**	**thickness**	**morpho**	**0.00021**	**0**	**0.00072**	**0**	**0.396**	**0.306**	**0.486**
**OK**	**IL**	**thickness**	**morpho**	**0.00041**	**0**	**0.00039**	**0**	**0.539**	**-0.812**	**1.889**
**OK**	**MN**	**thickness**	**morpho**	**0.00013**	**0**	**0.00025**	**0**	**0.510**	**0.510**	**0.510**

**IL**	**OK**	**thickness**	**morpho**	**0.00034**	**0**	**0.00049**	**0**	**0.000**	**0.000**	**0.000**
**IL**	**IL**	**thickness**	**morpho**	**0.00049**	**0**	**0.00036**	**0**	**0.000**	**0.000**	**0.000**
IL	MN	thickness	morpho	0.00007	0	0.00027	0	0.103	0.103	0.103
**MN**	**OK**	**thickness**	**morpho**	**0**	**0**	**0.00093**	**0**	**0.771**	**0.771**	**0.771**
**MN**	**IL**	**thickness**	**morpho**	**0.00048**	**0**	**0.0006**	**0**	**0.033**	**0.033**	**0.033**
**MN**	**MN**	**thickness**	**morpho**	**0.00014**	**0**	**0.00032**	**0**	**0.307**	**0.307**	**0.307**
OK	OK	ANT	physio	0	0	0.67939	0.12726	0.074	0.062	0.085
OK	IL	ANT	physio	0	0	28.08793	3.0288	0.090	-0.090	0.270
OK	MN	ANT	physio	0.94464	0.70602	1.50806	0.65116	0.205	0.205	0.205
IL	OK	ANT	physio	0.08032	0.16102	0.14833	0.15643	0.029	0.029	0.029
**IL**	**IL**	**ANT**	**physio**	**12.33**	**8.6856**	**22.5114**	**7.9701**	**0.428**	**0.101**	**0.754**
**IL**	**MN**	**ANT**	**physio**	**0.06006**	**0.0407**	**0.08039**	**0.0366**	**0.185**	**0.055**	**0.315**
MN	OK	ANT	physio	0.43886	0.55982	0.23574	0.51252	0.339	0.339	0.339
**MN**	**IL**	**ANT**	**physio**	**16.474**	**14.886**	**51.151**	**14.542**	**0.306**	**0.306**	**0.306**
**MN**	**MN**	**ANT**	**physio**	**0.06815**	**0.0558**	**0.15232**	**0.0044**	**0.386**	**0.386**	**0.386**
OK	OK	CCI	physio	0	0	0.00232	0	0.374	0.296	0.453
OK	IL	CCI	physio	0.00022	0	0.00187	0	0.435	-0.698	1.568
OK	MN	CCI	physio	0.00068	0.00141	0.00563	0.00141	0.302	0.302	0.302
IL	OK	CCI	physio	0.00001	0.001	0.00152	0.001	0.636	0.636	0.636
**IL**	**IL**	**CCI**	**physio**	**0.00089**	**0**	**0.00212**	**0**	**0.309**	**0.155**	**0.463**
**IL**	**MN**	**CCI**	**physio**	**0.00103**	**0.0014**	**0.00455**	**0.0014**	**0.225**	**0.225**	**0.225**
MN	OK	CCI	physio	0.00081	0.001	0.00032	0.001	0.185	0.185	0.185
**MN**	**IL**	**CCI**	**physio**	**0.00048**	**0**	**0.00183**	**0**	**0.134**	**0.134**	**0.134**
**MN**	**MN**	**CCI**	**physio**	**0.0006**	**0**	**0.00206**	**0**	**0.104**	**0.104**	**0.104**
OK	OK	WBI	physio	0	0	0.00001	0	0.693	0.675	0.710
OK	IL	WBI	physio	0	0	0.00003	0	0.339	-8.956	9.635
OK	MN	WBI	physio	0	0	0.00012	0	0.348	0.348	0.348
IL	OK	WBI	physio	0.00001	0	0	0	0.024	0.024	0.024

**IL**	**IL**	**WBI**	**physio**	**0.00029**	**0**	**0.00011**	**0**	**0.385**	**0.090**	**0.681**
**IL**	**MN**	**WBI**	**physio**	**0**	**0**	**0.0001**	**0**	**0.108**	**0.041**	**0.176**
MN	OK	WBI	physio	0	0	0.00013	0	0.000	0.000	0.000
**MN**	**IL**	**WBI**	**physio**	**0.00001**	**0**	**0.0002**	**0**	**0.000**	**0.000**	**0.000**
**MN**	**MN**	**WBI**	**physio**	**0.00001**	**0**	**0**	**0**	**0.000**	**0.000**	**0.000**
**OK**	**OK**	**R700**	**spectra**	**0.0002**	**0**	**0.00056**	**0**	**0.423**	**0.223**	**0.622**
**OK**	**IL**	**R700**	**spectra**	**0.00035**	**0**	**0.0001**	**0**	**0.025**	**-0.165**	**0.216**
OK	MN	R700	spectra	0	0	0.00219	0	0.410	0.410	0.410
IL	OK	R700	spectra	0	0	0.00051	0	0.909	0.909	0.909
**IL**	**IL**	**R700**	**spectra**	**0.00013**	**0**	**0.00023**	**0**	**0.351**	**-0.325**	**1.027**
**IL**	**MN**	**R700**	**spectra**	**0.00069**	**0**	**0.00134**	**0**	**0.005**	**-0.004**	**0.013**
MN	OK	R700	spectra	0	0	0.00053	0	0.000	0.000	0.000
MN	IL	R700	spectra	0	0	0.00052	0	0.000	0.000	0.000
**MN**	**MN**	**R700**	**spectra**	**0.00033**	**0**	**0.00146**	**0**	**0.539**	**0.539**	**0.539**
**OK**	**OK**	**R900**	**spectra**	**0.00036**	**0**	**0.00051**	**0**	**0.402**	**0.309**	**0.494**
**OK**	**IL**	**R900**	**spectra**	**0.00004**	**0**	**0.00126**	**0**	**0.000**	**0.000**	**0.000**
OK	MN	R900	spectra	0	0	0.00279	0	0.000	0.000	0.000
IL	OK	R900	spectra	0	0	0.00061	0	0.000	0.000	0.000
**IL**	**IL**	**R900**	**spectra**	**0.0001**	**0**	**0.00192**	**0**	**0.651**	**-0.784**	**2.085**
**IL**	**MN**	**R900**	**spectra**	**0.00026**	**0**	**0.0006**	**0**	**0.719**	**-1.060**	**2.498**
MN	OK	R900	spectra	0.00122	0.001	-0.00026	0.001	0.000	0.000	0.000
MN	IL	R900	spectra	0.00032	0	0.00116	0	1.267	-2.395	4.929
**MN**	**MN**	**R900**	**spectra**	**0.00009**	**0**	**0.00056**	**0**	**1.259**	**1.259**	**1.259**
**OK**	**OK**	**R1400**	**spectra**	**0.00039**	**0**	**0.00013**	**0**	**0.976**	**0.247**	**1.706**
**OK**	**IL**	**R1400**	**spectra**	**0.00019**	**0**	**0.00042**	**0**	**0.284**	**-0.760**	**1.327**
OK	MN	R1400	spectra	0	0	0.00105	0	0.227	0.227	0.227
IL	OK	R1400	spectra	0.00025	0	0.00021	0	0.000	0.000	0.000
**IL**	**IL**	**R1400**	**spectra**	**0.00024**	**0**	**0.00036**	**0**	**0.000**	**0.000**	**0.000**

**IL**	**MN**	**R1400**	**spectra**	**0.00015**	**0**	**0.00025**	**0**	**0.000**	**0.000**	**0.000**
MN	OK	R1400	spectra	0.00085	0	-0.00018	0	0.261	0.261	0.261
MN	IL	R1400	spectra	0.00012	0	0.00041	0	0.413	0.413	0.413
**MN**	**MN**	**R1400**	**spectra**	**0.00003**	**0**	**0.00029**	**0**	**0.754**	**0.754**	**0.754**

Figures

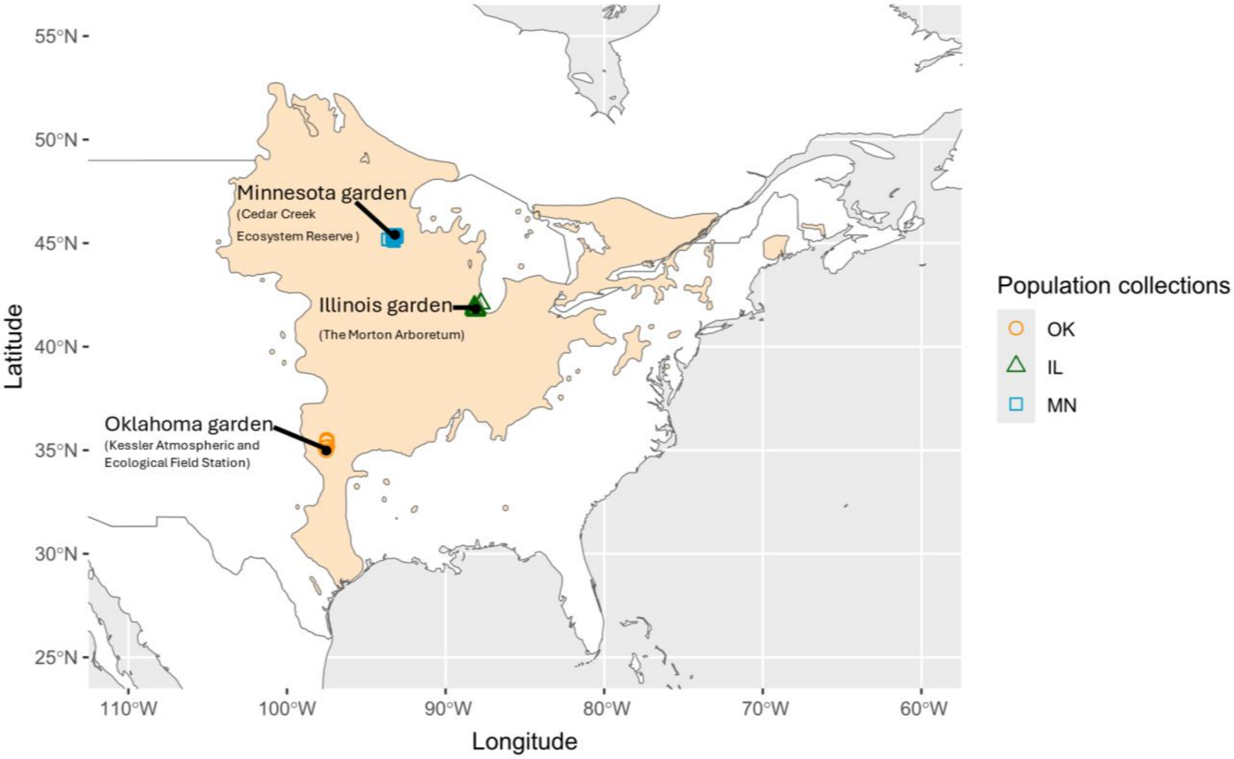

Figure S1. Map of collections for each population, overlayed over species distribution for *Quercus macrocarpa* from United States Geological Survey.

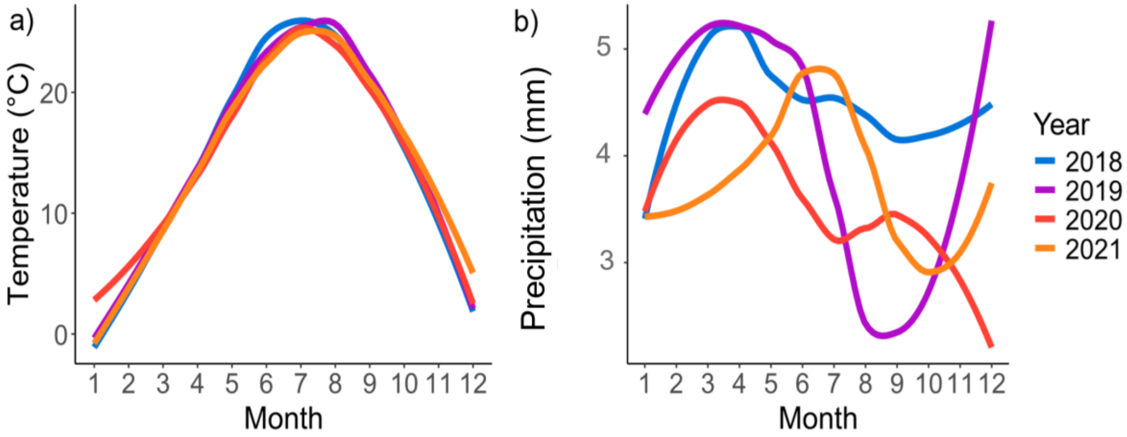

Figure S2. Temperature and precipitation in Vallonia, Indiana Department of Natural Resources Nursery, where trees were germinated and grew from 2018-2021. Climate data was acquired from ERA5. The colored lines indicate the trends for years 2018 (blue), 2019 (purple), 2020 (red) and 2021 (orange).

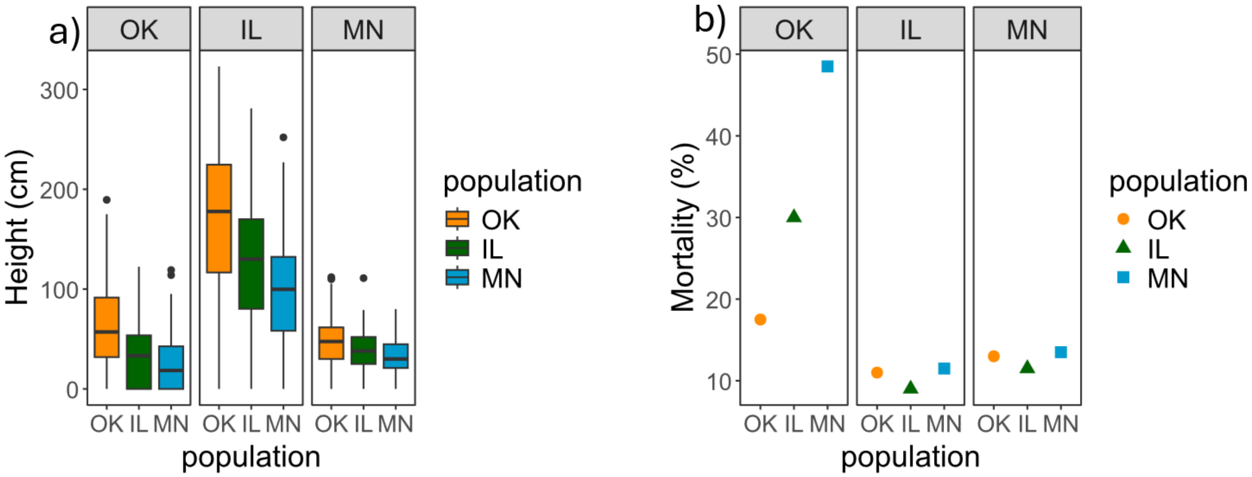

Figure S3. Box plot of heights for each population in each garden in 2023. Percent mortality in 2023 in each population in each garden.

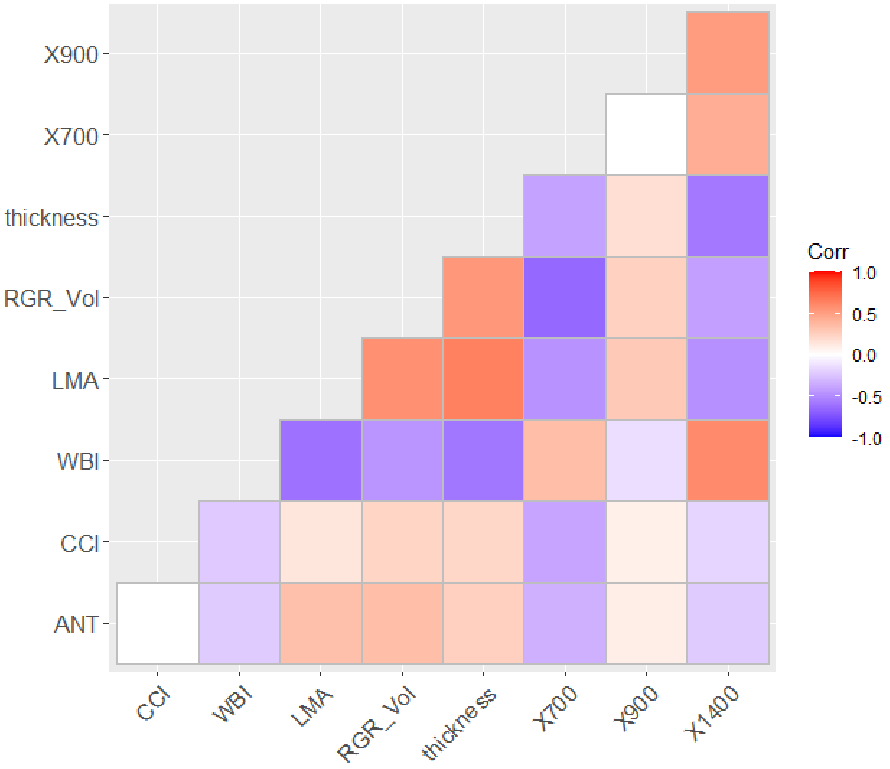

Figure S4. Correlation matrix of traits used in selection analysis.

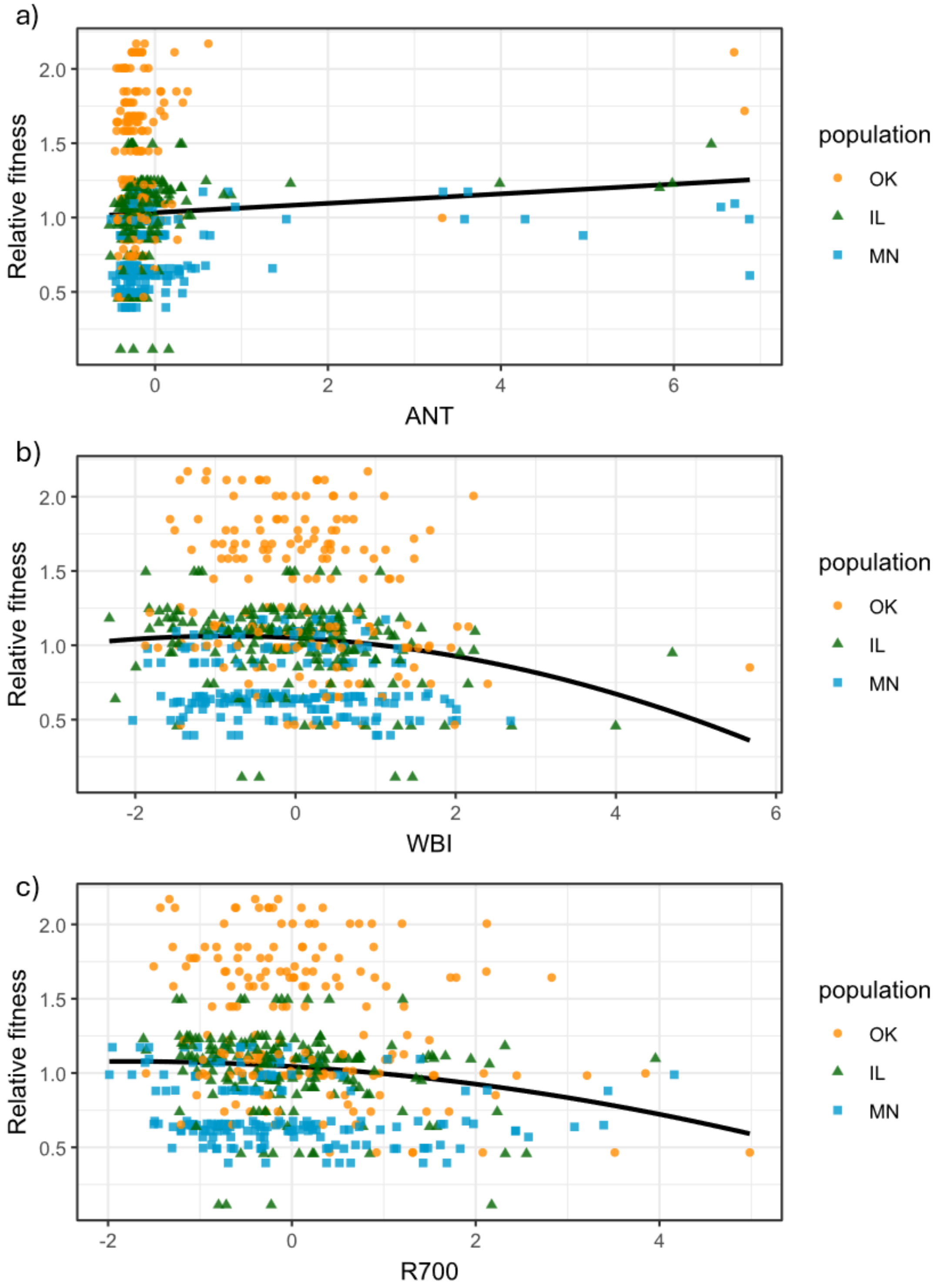

Figure S5. Two dimensional plots of quadratic selection on anthocyanin (ANT), water balance index (WBI), and spectral reflectance at 700 nm (R700) in the Illinois garden.
